# Pentabromopseudilin: a myosin V inhibitor suppresses TGF-**β** activity by recruiting the type II TGF-**β** receptor to lysosomal degradation 

**DOI:** 10.1080/14756366.2018.1465416

**Published:** 2018-05-16

**Authors:** Wang Shih-Wei, Chung Chih-Ling, Yu-Chen Kao, René Martin, Hans-Joachim Knölker, Meng-Shin Shiao, Chun-Lin Chen

**Affiliations:** a Department of Biological Sciences, National Sun Yat-sen University, Kaohsiung, Taiwan, ROC;; b Department of Chemistry, TU Dresden, Dresden, Germany;; c Faculty of Medicine Ramathibodi Hospital, Mahidol University, Bangkok, Thailand;; d Doctoral Degree Program in Marine Biotechnology, National Sun Yat-sen University and Academia Sinica, Kaohsiung, Taiwan, ROC

**Keywords:** Myosin V, pentabromopseudilin, subcellular trafficking, lipid-raft, TGF-*β*

## Abstract

Pentabromopseudilin (PBrP) is a marine antibiotic isolated from the marine bacteria *Pseudomonas bromoutilis* and *Alteromonas luteoviolaceus*. PBrP exhibits antimicrobial, anti-tumour, and phytotoxic activities. In mammalian cells, PBrP is known to act as a reversible and allosteric inhibitor of myosin Va (MyoVa). In this study, we report that PBrP is a potent inhibitor of transforming growth factor-β (TGF-β) activity. PBrP inhibits TGF-β-stimulated Smad2/3 phosphorylation, plasminogen activator inhibitor-1 (PAI-1) protein production and blocks TGF-β-induced epithelial–mesenchymal transition in epithelial cells. PBrP inhibits TGF-β signalling by reducing the cell-surface expression of type II TGF-β receptor (TβRII) and promotes receptor degradation. Gene silencing approaches suggest that MyoVa plays a crucial role in PBrP-induced TβRII turnover and the subsequent reduction of TGF-β signalling. Because, TGF-β signalling is crucial in the regulation of diverse pathophysiological processes such as tissue fibrosis and cancer development, PBrP should be further explored for its therapeutic role in treating fibrotic diseases and cancer.

## Introduction

**Figure 1.**
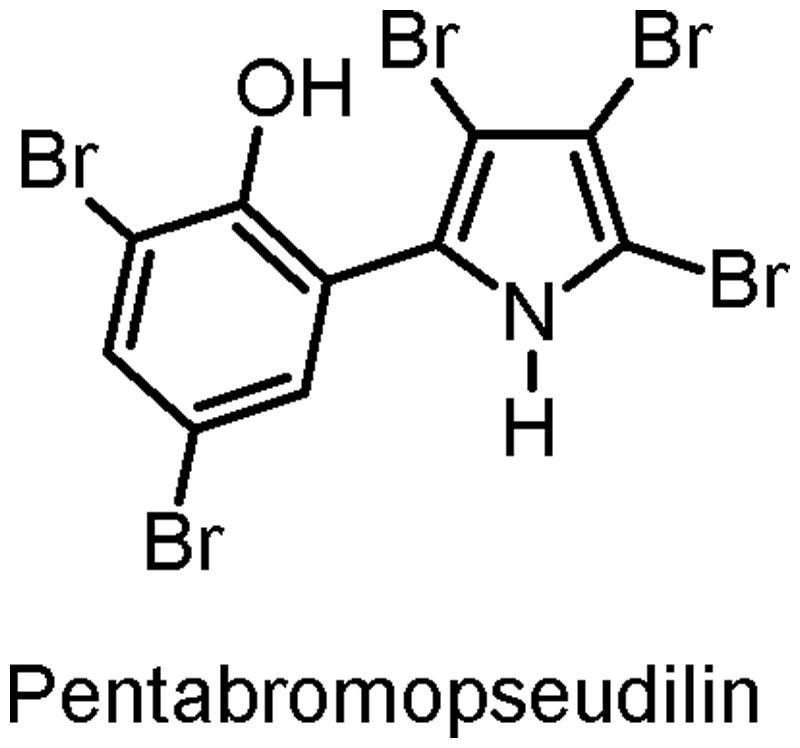
Structure of pentabromopseudilin.

Pentabromopseudilin [2,3,4-tribromo-5-(3,5-dibromo-2-hydroxyphenyl)-1*H*-pyrrole] (PBrP; Figure 1) is a potent antibiotic originally isolated from the marine bacterium *Pseudomonas bromoutilis*^1^. PBrP exhibits various biological (i.e. cytotoxic, antibacterial and phytotoxic) activities, including the inhibition of human 12- and 15-lipoxygenases^2^ and potent inhibition of myosin-dependent processes^3–5^. Recently, PBrP was identified as a potent inhibitor of the motor activity of vertebrate myosin V with an IC_50_ value of 1.2 μM^5^. PBrP inhibits the ATPase activity of this myosin by increasing its affinity for ADP, reducing ATP-binding and hydrolysis rates, and through the coupling between actin- and nucleotide-binding sites in the motor domain^3^. In this study, we demonstrate that PBrP, as a specific inhibitor of transforming growth factor-β (TGF-β), suppresses TGF-β-induced cellular responsiveness by displacing type II TGF-β receptor (TβRII) from the plasma membrane into the lysosomal degradation pathway.

TGF-β is a member of a large family of evolutionarily conserved pleiotropic cytokines that also includes bone morphogenetic proteins, activins and nodals. TGF-β is crucial for the regulation of various biological processes during embryogenesis and adult tissue homeostasis. Dysregulation of TGF-β signalling has been associated with many diseases such as tissue fibrosis and cancer. TGF-β exerts protective or tumour suppressive effects on normal epithelial cells and during the early growth-sensitive stages of tumorigenesis. However, in the late stages of malignancy, tumour progression is driven by a TGF-β overload. The canonical pathway of TGF-β signalling is triggered by the binding of the TGF-β to TβRII and promotion of the formation of a receptor heterocomplex, where TβRII phosphorylates type I TGF-β receptor (TβRI). Activated TβRI phosphorylates receptor-activated Smads (R-Smads: Smad2 and Smad3), which subsequently form a heterocomplex with Smad4. The Smad complex is then rapidly translocated to the nucleus to regulate target gene activity.

The output of conserved signal transduction pathways, including TGF-β-mediated signalling and those mediated by the epidermal growth factor receptor (EGFR), Notch and G protein-coupled receptors, depends not only on the activation of these receptors by extracellular stimuli, but also on the endocytic internalisation and postendocytic trafficking of receptors, which regulate the availability and compartmentalisation of the signal transduction machinery^6–8^. TGF-β receptors are compartmentalised between lipid-raft and non-raft membrane microdomains, where they can be endocytosed by caveolae/lipid-raft and clathrin-coated vesicles, respectively. Clathrin-mediated endocytosis brings TGF-β receptors to early endosome antigen-1 (EEA-1)-positive endosomes and likely promotes R-Smad activation^9^^,^^10^. The internalised receptors can be returned to the plasma membrane through Rab-11-mediated exocytosis^11^. However, in our previous study, we demonstrated that the inhibitors of clathrin-dependent endocytosis, which also arrest the progression of endocytosis in coated-pit stages, inhibit the internalisation of cell-surface-bound TGF-β and sustain the colocalisation and accumulation of TβRI and the Smad anchor for receptor activation (SARA) in the plasma membrane, resulting in enhanced TGF-β-induced signalling and responses^12^. This study suggests that the number of surface receptors is also a decisive factor for cellular responses to TGF-β stimulation. Internalisation of TGF-β receptors through lipid-raft/caveolae-mediated endocytosis is more likely to facilitate receptor degradation through lysomal/proteasomal degradation pathways, thereby turning off TGF-β signalling. In line with this finding, we observed that triterpenoids and cholesterol derivatives suppress TGF-β responsiveness in cultured cells by causing the accumulation of cell-surface-bound TGF-β–TGF-β receptor complexes in the lipid rafts/caveolae of the plasma membrane, thereby facilitating the rapid degradation of these complexes, and thus attenuating TGF-β-stimulated signalling and -related responses^13–15^. The data obtained in this study reveal that PBrP suppresses the TGF-β signalling pathway, as determined using a 3TP-Lux plasmid [construct operated by the plasminogen activator inhibitor-1 (PAI-1) promotor] in a luciferase assay and observing Smad2/3 phosphorylation and their subsequent nuclear translocation. In human lung adenocarcinoma (A549) cells, PBrP potently attenuated TGF-β-induced epithelial–mesenchymal transition (EMT), including the reduction of cell migration and reduced expression of EMT-related genes such as N-cadherin, vimentin and fibronectin. Based on these results that confirm the role of PBrP in TGF-β receptor exocytosis/recycling and rapid degradation, we hypothesise that PBrP facilitates the accumulation of TGF-β receptors in the cytoplasm by inhibiting the recycling of TGF-β receptors to the cell surface, leading to lysosomal degradation and subsequent decrease in TGF-β signalling. TGF-β signalling pathways are key mediators that control proliferation and inflammation, and are regulated by endocytosis and exocytosis. Therefore, the present findings may provide a cell biological basis for potential therapeutic applications of motor protein inhibitors to treat various TGF-β-related diseases.

## Materials and methods

### Compounds

PBrP (Figure 1) was synthesised and provided by Dr. Hans-Joachim Knölker from TU Dresden (Bergstrasse, Germany)^16^. PBrP was dissolved in DMSO as aliquoted stock (10 mM) and then stored at −80 °C. The final concentrations of DMSO in all experiments were lower than 0.1%, and thus DMSO had no effect on TGF-β signalling^17^.

### Stable cell lines

Mv1Lu, A549, HepG2, Clone 9 and HEK293 cells were obtained from ATCC (Manassas, VA). The PAI-1 promotor stable clone of Mv1Lu cells (MLECs-Clone 32) was a gift from Dr. Jung San Huang from Saint Louis University. The cells were cultured at 37 °C in 5% CO_2_ in Dulbecco’s modified Eagle’s medium (DMEM; Gibco, Waltham, MA, USA) containing 5% FBS (Life Technologies, Waltham, MA, USA) with 50 μg/mL of streptomycin. To generate a stable MyoVa deficiency cell line, HEK293T cells were used to produce indicated lentiviral particles through the viral packaging process. The supernatant containing the virus was collected and filtered. Subsequently, A549 cells were infected with lentiviral particles. Lentiviral particles containing MyoVa (TRCN0000018214) or control shRNA lentiviral particles (TRCN0000018214) were purchased from the National RNAi Core Facility of Academic Sinica (Taiwan). The infected A549 cells were selected using 2–4 μg/mL of puromycin for 2 weeks.

### Materials

Alexa Fluor 488- and 594-conjugated secondary antibodies and DAPI were purchased from ThermoFisher (Waltham, MA, USA). TGF-β was obtained from PeproTech (Rocky Hill, NJ, USA). A prestained protein ladder (125, 93, 72, 57, 42, 31, 24, and 15 kDa) was purchased from GeneDireX (Carlsbad, CA, USA). Trichloroacetic acid (TCA), phenylmethanesulfonyl fluoride (PMSF), CHX, MG132, trifluoperazine, NH_4_Cl, and methyl-β-cyclodextrin were purchased from Sigma-Aldrich (St. Louis, MO, USA). Concanamycin A (CA) and chloroquine (CQ) were purchased from Cayman Chemical (Ann Arbor, MI, USA). The COL1A2-luc plasmid was constructed as described by Poncelet et al.^18^, and the Fibro-luc plasmid was constructed as described by Cobbs and Widom^19^^,^^20^. TβRII-flag (#31719), RFP-rab5 (#14337), GFP-rab11 (#12647), and Lamp-1 (#1816) plasmids were purchased from Addgene (Cambridge, MA, USA). TRIzol reagent was purchased from Invitrogen (Carlsbad, CA, USA). M-MLV reverse transcriptase was obtained from Promega (Madison, WI, USA).

### Western blotting and antibodies

To examine the effect of PBrP on TGF-β-induced Smad2/3 phosphorylation, Mv1Lu and A549 cells were incubated with PBrP at various concentrations or for various periods and then stimulated with 100 pM of TGF-β for 30 min. To monitor EMT protein expression, the cells were pretreated with different PBrP concentrations for 2 h and then stimulated with TGF-β for 48 h. The cells were lysed with a lysis buffer and standardised using a bicinchoninic acid (BCA) protein assay (Pierce™ BCA Protein Assay Kit 23225). Fifty microgram of this protein was applied to SDS-PAGE and electrotransferred to the polyvinylidene difluoride (PVDF) membrane. Polyclonal antibodies against TβRI (sc-398), TβRII (sc-400), EGFR (sc-373746), β-actin (sc-47778), flotilin-1 (sc-25506), fibronectin (sc-9068), N-cadherin (sc-7939), Lamin B (sc-6216) and vimentin (sc-7557) were obtained from Santa Cruz (Dallas, TX). Rabbit polyclonal antibodies against pSmad2/3 (#8828), Smad2/3 (#8685), PAI-1 (#11907), Flag tag (#8146), Lamp-1 (sc-9091) and MyoVa (#3402) were purchased from Cell Signalling (Boston, MA). Secondary antibodies conjugated with horseradish peroxidase (Millipore, Darmstadt, Germany) and an enhanced chemiluminescence (ECL) kit (Perkin-Elmer Life Sciences, Waltham, MA, USA) were used to develop immunoblots.

### Luciferase assays

TGF-β-stimulated luciferase activity assay was conducted following a previous report^15^^,^^21^^,^^22^. MLECs were grown to 80% confluency on a 24-well cluster plate and then pretreated with several concentrations of PBrP or vehicle only (in DMEM containing 0.1% FBS) for 2 h, followed by stimulation with 100 pM of TGF-β for 4 h. In similar experiments, Mv1Lu cells were transiently transfected with CMV-β-gal and Fibro-luc or COL1A2-luc reporter plasmids through electroporation (Gene Pulser Xcell, BioRad, Hercules, CA, USA). After 4 h, treated cells were lysed in 100 μL of lysis buffer (Promega) at 37 °C. The TGF-β-stimulated luciferase activity of cell lysates (20 μg of protein) was subsequently assayed using the luciferase kit from Promega. Cells treated without exogenous TGF-β exhibited a basal level of luciferase activity, which was stimulated by endogenous TGF-β.

### shRNA silencing and lentiviral transduction

MyoVa and control shRNA were obtained from the National RNAi Core Facility (Institute of Molecular Biology, Academia Sinica, Taipei, Taiwan). The target sequences used for MyoVa siRNA1, MyoVa siRNA2 and control were 5′-CCGGCGGATTTGAAACATT-TGAGATCTCGAGATCTCAAATGTTTCAAATCCGTTTTTG-3′, 5′-CCGGCGCTTTATTGATTCCAAACTTCTCGAGAAGTTTGGAATCAATAAAGCGTTTTTG-3′ and 5′-CATCCGAAGCCACACACTG-3′, respectively. Control and MyoVa shRNA lentiviruses were produced by co-transfecting plasmids (5 μg of scrambled or MyoVa shRNA and 10 μg of lentivirus packaging vectors) into 293T cells (Invitrogen) using Lipofectamine 3000. After 24-h exposure to the transfection mixture, the medium was collected. After another 24 h, the virus-containing supernatant was harvested again. Two batches of the supernatant were centrifuged to remove cell debris and used to infect A549 cells in the presence of 8 μg/mL of polybrene (Sigma-Aldrich). After 48-h infection, selection was performed using 5 μg/mL of puromycin. Puromycin-resistant clones subsequently were pooled and used for experiments.

### Analysis of lipid raft/caveolae and lipid non-raft microdomains

Membrane lipid rafts were purified and analysed using sucrose density gradient fractionation^17^. The purified cellular plasma membrane was solubilised in 0.85 ml of 500-mM sodium carbonate in three 15-s bursts of an ultrasonic disintegrator. The membrane lysates were adjusted to 45% sucrose by adding 0.85 ml of 90% sucrose in 2-(N-morpholino) ethanesulfonic acid (MES)-buffered saline (MBS; 25 mM of MES, pH 6.5, 0.15 M of NaCl). Subsequently, 1.7 ml of the extraction mixture was placed at the bottom of an ultracentrifuge tube and 1.7 ml of 35% sucrose and 1.7 ml of 5% sucrose in MBS were overlaid^23^. The gradient was generated in a SW55Ti rotor (Beckman, Brea, CA, USA) at 40,000 rpm for 20 h at 4 °C. Ten 0.5-ml fractions were collected from the top of the tube and concentrated through TCA precipitation, and the protein sediments of each fraction were further subjected to SDS-PAGE and Western blotting. The relative amounts of TβRII on the blot were quantified through densitometry. Fractions 4–5 and 7–10 contained flotillin-2 and EEA-1, respectively^15^^,^^17^.

### Cell-surface receptor biotinylation and endocytosis assays

Cell-surface biotinylation was performed at 0 °C using water-soluble membrane impermeable sulfo-NHS-SS-biotin (ThermoFisher) following the manufacturer’s instructions and a reported procedure^24^. Mv1Lu cells grown to 90% confluence on six-well cluster dishes were treated with PBrP for various periods at 37 °C. After treatment, the cells were rinsed twice with cold phosphate-buffered saline (PBS) supplemented with 0.5 mM of MgCl_2_ and 1 mM of CaCl_2_ prior to the exposure of cells to 0.2 mM of sulfo-NHS-SS-biotin. After biotinylation, to remove all unreacted biotin, biotinylated cells were washed with a quenching agent containing 50 mM of glycine and 0.5% of BSA dissolved in PBS. Subsequently, the cells were lysed in a lysis buffer and the biotinylated proteins were captured from the supernatant using affinity chromatography with NeutrAvidin slurry at 4 °C for 2 h. The slurry was then boiled in Laemmli gel-loading buffer for 5 min prior to 10% SDS-PAGE, followed by immunoblotting analysis and quantification with ImageQuant (Chicago, IL, USA). Biotinylated TβRII remaining on the cell surface was compared with the total TβRII level without biotinylation.

### Immunofluorescence staining

To determine the effect of PBrP on the subcellular localisation of TβRII, HEK293 cells grown on 24-mm round coverslips were transiently cotransfected with TβRII-Flag and caveolin-1-GFP or rab5, rab9, rab11, and Lamp-1 plasmids by using Lipofectamine 3000 (ThermoFisher), following the manufacturer’s protocol. Twenty-four hours after transfection, the cells were serum starved and treated with 5 μM of sorafenib for the time indicated. After treatment, the cells were fixed with 4% paraformaldehyde solution containing 0.1% of Triton-X 100 for 30 min, washed with PBS, and then blocked using 0.2% gelatin in PBS for 1 h. The cells were incubated overnight at 4 °C in a humidified chamber with a goat anti-flag-probe at 1:100 dilution. After extensive washing, the cells were incubated using the Alexa Fluor 594-conjugated donkey anti-goat antibody at 1:50 dilution for 1 h. The coverslips were mounted using a mounting medium containing DAPI. Images were captured using a Nikon TCS SP confocal microscope (Nikon Ltd., Tokyo, Japan).

### Scratch assay and random migration movies

The scratch assay was analysed through life cell imaging, which was performed using an Axio Observer Z1 inverted microscope (Zeiss, Oberkochen, Germany), and time-lapse pictures were taken with a CoolSNAP HQ2 Monochrome camera (Photometrics, Tucson, AZ, USA) in a live-cell environmental chamber (Pecon, Oberkochen, Germany). A549 cells were grown to confluence on a 24-well cluster tissue culture plate (Nunc™, Waltham, MA, USA) and scratched with a p200 pipette tip. The extent of cell migration was measured using the difference in the width of the scratch between 0 h and the final time point. To generate videos, the inverted microscope was fitted with an atmosphere chamber (37 °C and 5% of CO_2_). Bright-field images were obtained at 100 × every 20 min over a 24-h period. Movies were then analysed from these snapshots in ImageJ (Bethesda, MD, USA).

### Data analysis

All experiments were conducted in triplicate. All data are presented as mean ± standard deviation (SD), and statistical analyses were performed using SPSS software (IBM Corporation, Armonk, NY, USA). We conducted student’s *t*-test to compare the two groups and a one-way analysis of variance for comparison involving other groups. The means were considered significant if *p < .05 or **p < .01.

## Results

### PBrP prevents TGF-β-induced Smad protein phosphorylation and nuclear translocation

Smad2/3 proteins are the primary signal transducers in TGF-β signalling. After stimulation with TGF-β, phosphorylated Smad2/3 and Smad4 form a complex and co-migrate to the nucleus. The inhibitory effects of PBrP on TGF-β-induced Smad2/3 phosphorylation were evaluated in Mv1Lu, A549, HepG2, and Clone9 cells through immunoblotting. The cells were preincubated with increasing PBrP concentrations for 6 h and continuously stimulated with TGF-β (20 or 100 pM) for 0.5 h. A representative Western blot of phospho-Smad (pSmad) 2/3 and Smad2/3 is shown in Figure 2. TGF-β strongly stimulates the phosphorylation of Smad2/3 proteins in Mv1Lu, A549 and HepG2 cell lines (lane 7 versus 1), whereas PBrP treatment suppresses the TGF-β-stimulated Smad2/3 phosphorylation in a dose-dependent manner in these cell lines (Figure 2(A–C)). Although the concentration ranges of PBrP were different, Mv1Lu and A549 cells exhibited similar sensitivity to PBrP with an IC_50_ value of approximately 0.1 μM. However, HepG2 cells exhibited a lower sensitivity to PBrP with an IC_50_ value of approximately 0.2 μM. To examine the chronological fashion of PBrP inhibitory effects, the cells were pretreated with 0.2 or 0.5 μM PBrP for increasingly long periods (0.5, 1, 2, 4 and 6 h) followed by TGF-β stimulation. The treatment of cells with PBrP blocked TGF-β-induced Smad2/3 protein phosphorylation in a time-dependent manner, and PBrP rapidly reduced >90% of pSmad2/3 signalling in 6 h of treatment (Figure 2(D–G)). A549 cells were used to determine the inhibitory effect of PBrP on the TGF-β-induced nuclear translocation of Smad proteins. The results of Western blotting, which was performed to analyse nuclear fractions after treating cells with TGF-β and PBrP, revealed that TGF-β induced the nuclear translocation of Smad2/3 within 30 min and this nuclear translocation was prevented by pretreatment with PBrP (Figure 2(H) and relative graph). PBrP alone did not alter the localisation of Smad proteins. The nuclear protein Lamin B was used as a loading control and to confirm nuclear separation. To further define the subcellular translocation of endogenous Smad2/3, A549 cells were pretreated with 0.2 μM of PBrP or a suitable amount of dimethyl sulfoxide (DMSO) for 6 h, then stimulated with TGF-β for 0.5 h, and finally analysed through immunofluorescent staining using an antibody against Smad2/3. Upon TGF-β stimulation, the immunofluorescent stain was found to be more concentrated in the nuclei, indicating that the nuclear translocation of Smad2/3 occurred in response to TGF-β stimulation. However, as shown in Figure 2(I), PBrP abolished TGF-β-induced Smad2/3 nuclear translocation (Figure 2(Ic,Ib)) and DMSO treatments did not alter the subcellular distribution of Smad2/3. These results suggest that PBrP prevents TGF-β-induced Smad2/3 activation and the subsequent nuclear translocation of Smad2/3.

**Figure 4.**
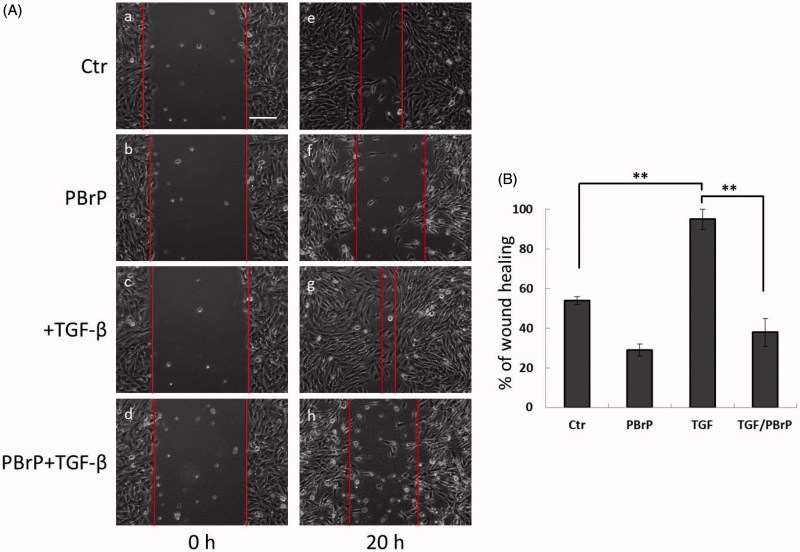
PBrP delays TGF-*β*-induced cell migration. Confluent monolayers of A549 cells in a 24-well cluster tissue culture plate were scratched and incubated at 37 °C for 20 h in DMEM containing 0.2% of FBS (Control; top). Cell motility was measured using a fully automatic microscope at 10× phase objective. Cell migration was observed by performing time-lapse microscopy and images of all four experimental conditions were captured simultaneously every 20 min. The red lines indicate the starting point of cell migration. Representative micrographs from three experiments are shown in panel (A). Right graphs illustrate of quantitative analyses of cell covering area (mean ± SD) from three independent experiments are shown (B) (% of TGF-*β* treatment alone); **p < .01. Bar: 10 *μ*m.

Figure 2.PBrP inhibits TGF-*β*-stimulated Smad2/3 phosphorylation and nuclear translocation. Mv1Lu (A), A549 (B) and HepG2 (C) cells were pretreated with increasing concentrations (0.01–1 *μ*M) of PBrP for 6 h followed by 30 min of TGF-*β* stimulation. Mv1Lu (D), A549 (E), HepG2 (F) and Clone 9 (G) cells were pretreated with 0.5 *μ*M of PBrP for 0.5, 1, 2, 4 and 6 h followed by 30 min of TGF-*β* stimulation. Cell lysates were subjected to Western blot analysis with monoclonal antibodies against p-Smad2/3 and Smad2/3. An equal amount of protein loading was verified by Smad2/3 and *β*-actin (images were duplicated from the experiments illustrated in Figure 4). (H) Nuclear translocation of Smad proteins was analysed by separating nuclear fractions after treating A549 cells with TGF-*β* or PBrP for 6 h. Smad2/3 and pSmad2/3 proteins were detected through Western blot analysis using antibodies against Smad2/3 and p-Smad2/3. Complete fractionation of nuclear proteins and equivalent loading were verified through Western blot analysis using antibodies against Lamin B. Panels (A to H) show the representative images and right graphs illustrate quantitative analyses of ECL (mean ± SD) from at least three independent experiments (compare with TGF-*β* treatment alone); *p < .05, **p < .01. (I) Blockade of TGF-*β*-induced nuclear import of Smad2/3 by PBrP in A549 cells was determined through immunofluorescence. Cells were serum starved and pretreated with 0.5 *μ*M of PBrP for 6 h followed by 30 min of TGF-*β* stimulation, and processed for immunofluorescence using the anti-Smad2/3 antibody. Fluorescence was visualised using the Alexa Fluor 488-conjugated donkey anti-goat antibody and a ZeissObserver fluorescence microscope. DAPI was used as a counterstain. Each experiment was repeated three times. Bar: 20 *μ*m.

**Figure:**
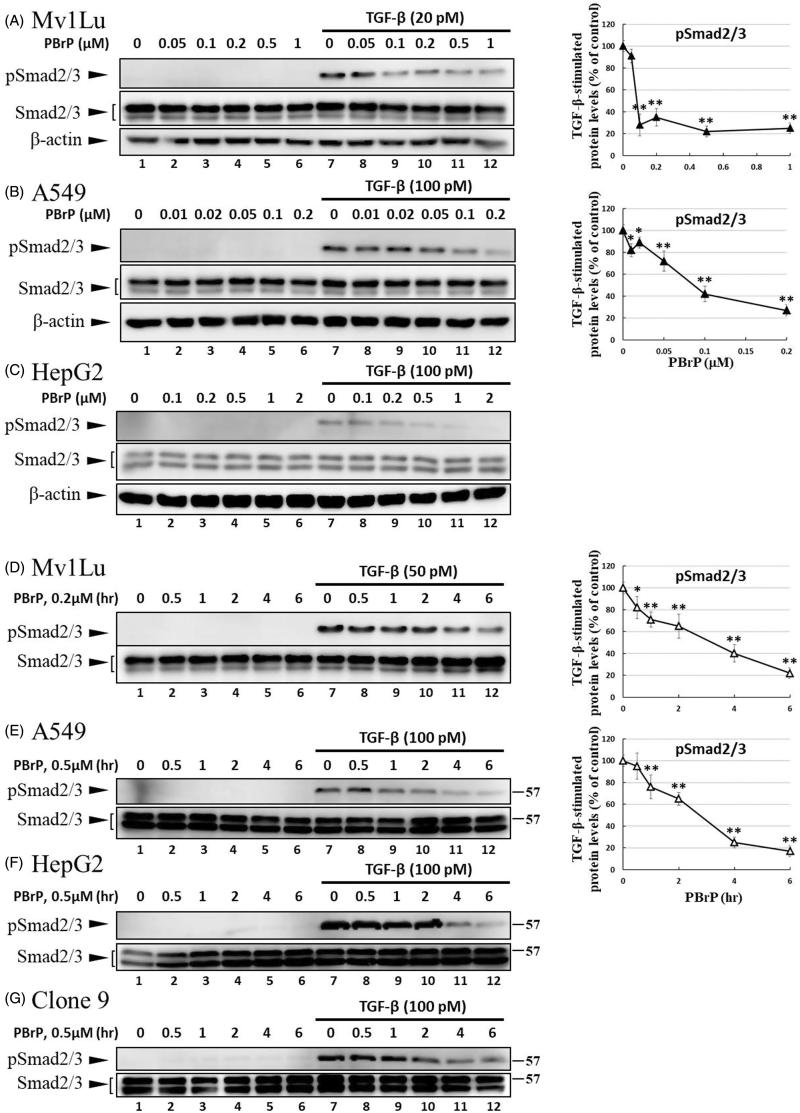

**Figure:**
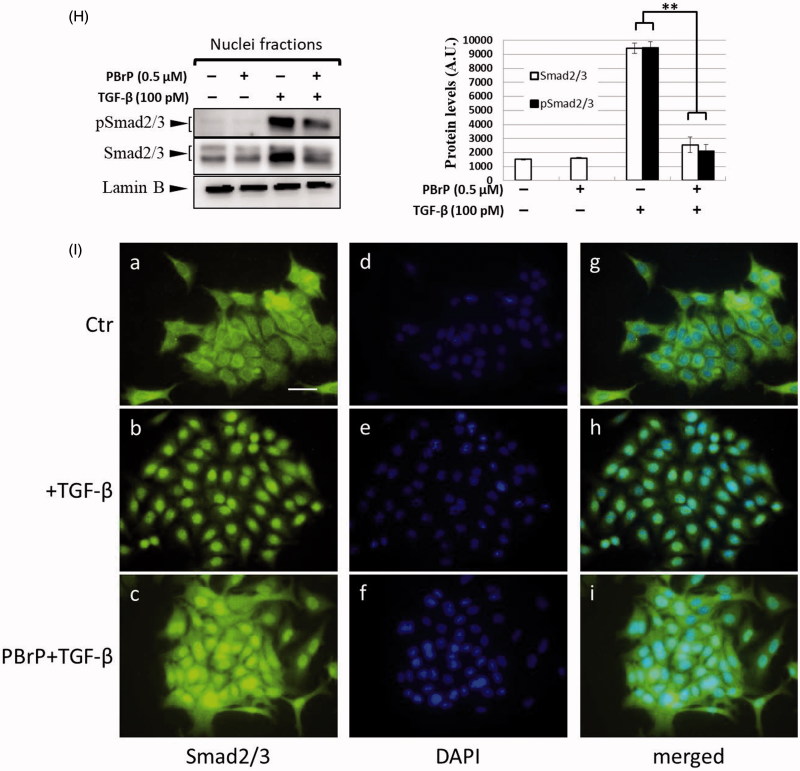

### PBrP inhibits TGF-β-stimulated transcriptional responses

To further define the downstream transcriptional responses mediated by TGF-β, we employed Mink lung epithelial cells (MLECs-Clone 32) that stably expressed the *PAI-I* gene, which was fused to the firefly luciferase reporter. TGF-β stimulation strongly induced luciferase activity and PBrP suppressed the TGF-β-induced transcriptional activation in a dose-dependent manner with an IC_50_ value of approximately 0.1 μM; the maximum inhibition achieved was 90–95% at 1 μM in each test (Figure 3(A)). In the positive control experiment, SB-431542 also inhibited TGF-β-induced transcriptional responses with a higher IC_50_ value of approximately 1 μM. Compared with TβRI kinase inhibitor SB431542, the PBrP exhibited a superior inhibitory effect in PAI-1 promoter assays (Figure 3(A)) and lower cytotoxicity in A549 cells (Supplemental Figure 1). To evaluate whether PBrP inhibits other TGF-β relative promoters such as fibronectin and collagen, A549 cells transiently expressing the *COL1A2-luc* or *Fibro-luc* reporter construct were pretreated with increasing PBrP concentrations, stimulated with TGF-β and subjected to luciferase assays. After normalisation through β-galactosidase activity, the A549 cells treated with PBrP demonstrated considerable reductions in TGF-β-stimulated activation of fibronectin and collagen (Supplemental Figure 2) in a dose-dependent manner with an IC_50_ value of approximately 0.1 μM, suggesting that PBrP is an effective inhibitor of the TGF-β signalling pathway.

Figure 3.PBrP blocks TGF-*β*-induced transcriptional responses and EMT protein production. (A) Inhibition of PAI-1 gene promoter-luciferase (PAI-1-Luc) activity in MLECs through a dose response of PBrP and the T*β*RI kinase inhibitor SB-431542 (SB) in response to TGF-*β*. (B to E) Control A549 cells (top) or cells pretreated with 0.5 *μ*M of PBrP for 6 h followed by 42 h of TGF-*β* stimulation were fixed and permeabilised. (B) Cells were incubated with TRITC-phalloidin (red) and DAPI (blue) to visualise the actin cytoskeleton and the nuclei, respectively. To visualize ZO-1 (C), fibronectin (D) and N-cadherin (E), cells pretreated with 0.5 *μ*M of PBrP for 6 h followed by 42 h of TGF-*β* stimulation were stained with specific antibodies and Alexa Fluor 488-conjugated secondary antibodies. Representative micrographs from three experiments are shown. Bar: 20 *μ*m. For Western blot analysis, A549 (F) and HepG2 (G) cells were treated with increasing doses of PBrP in DMEM containing 0.1% of FBS for 6 h and continually stimulated with or without 100 pM of TGF-*β* for 48 h. (H) A549 cells were pretreated with 0.5 *μ*M of PBrP for 6 h and continually stimulated with 0, 10 or 100 pM of TGF-*β* for 42 h. Cell lysates were then analysed through Western blotting with desired antibodies as indicated. The representative images (F to H) and right graphs illustrate of quantitative analyses of ECL (mean ± SD) from three independent experiments are shown (% of TGF-*β* treatment alone); *p < .05, **p < .01.

**Figure:**
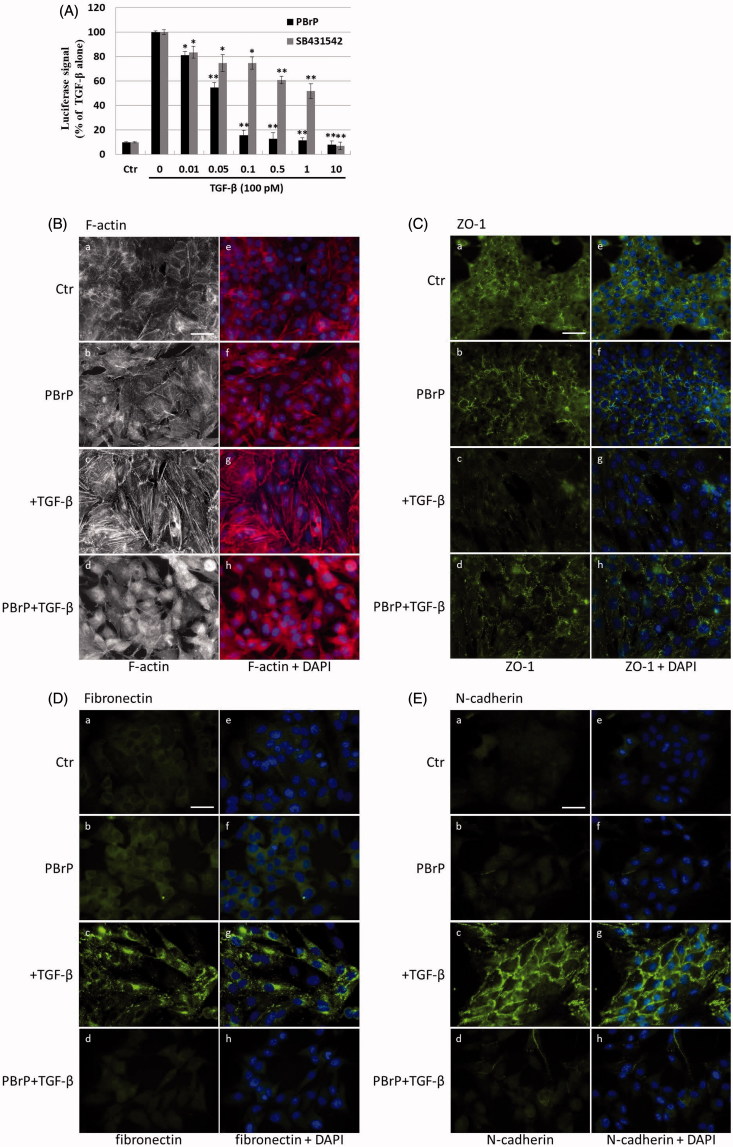

**Figure:**
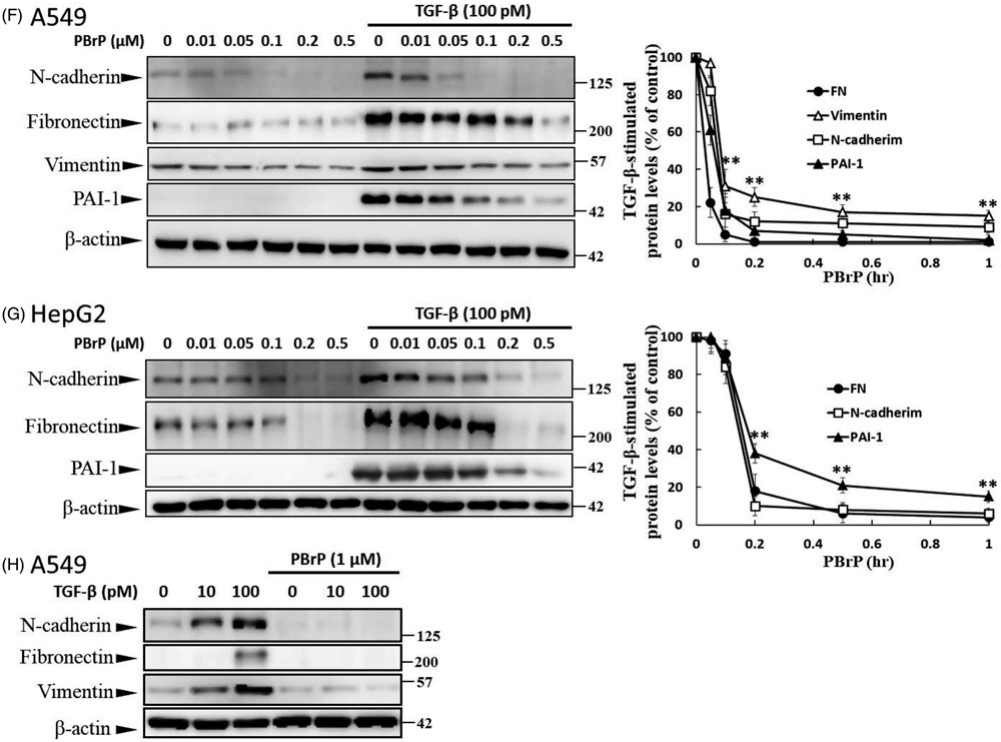

### PBrP inhibits TGF-β-induced EMT in A549 cells

The increased expression and activation of TGF-β in A549 and HepG2 cells markedly induced the progression of EMT^25^. EMT is characterised by the loss of cell polarity, disassembly of epithelial cell–cell junctions, reorganisation of the actin cytoskeleton with the formation of stress fibres and focal adhesions, acquisition of a spindle-shaped morphology, elevated expression of N-cadherin and delocalisation of E-cadherin from cell junctions. To determine whether PBrP affects TGF-β-stimulated EMT, we performed immunofluorescent staining to observe the subcellular localisation of F-actin, ZO-1, fibronectin and N-cadherin (Figure 3(B–E)). In the control and PBrP-alone treatments, F-actin was mainly distributed close to the plasma membrane (Figure 3(Ba)). TGF-β treatment altered the cellular ultrastructure, substantially increasing the cytoskeletal stress fibres distributed in the cells. The TGF-β-mediated effect was completely inhibited by PBrP treatment (Figure 3(Bd)). In addition, the cells under the control and PBrP-alone treatments exhibited ZO-1 distribution predominantly in the plasma membrane (Figure 3(Ca,Cb)), whereas fibronectin and N-cadherin levels were extremely low (Figure 3(Da,Ea)). TGF-β treatment dislocated ZO-1 from the plasma membrane and substantially increased the accumulation of fibronectin and N-cadherin in the cells (Figure 3(Dc,Ec)). PBrP treatment reversed the effect of TGF-β, as indicated by the reappearance of ZO-1 (Figure 3(Cd)) and the reduction of fibronectin and N-cadherin levels in A549 (Figure 3(Dd,Ed)) cells. Western blot analysis confirmed that the TGF-β-stimulated expression of N-cadherin, fibronectin, vimentin and PAI-1 proteins is abolished by PBrP in a dose-dependent manner in A549 and HepG2 cells, with IC_50_ values ranging from 0.01 to 0.2 μM (Figure 3(F,G)). Upon stimulation with two TGF-β concentrations (10 and 100 pM), N-cadherin, vimentin and fibronectin are completely eradicated by PBrP (Figure 3(H)) in A549 cells. These results suggest that PBrP plays a pivotal role in suppressing TGF-β-induced EMT.

### PBrP suppresses TGF-β-induced cell migration

We investigated the effects of PBrP on blocking TGF-β-stimulated cell mobility by using a wound-healing assay and A549 cells, which exhibited responsiveness to TGF-β in wound-healing experiments^21^. As shown in Figure 4, TGF-β increased cell mobility and promoted wound closure, whereas 0.2 μM of PBrP suppressed TGF-β-stimulated cell migration and did not close the wound (Figure 4(Ah)). A previous study suggested that A549 cells are growth inhibited by TGF-β, indicating that wound closure in these cells is not due to proliferation. To confirm that the inhibition of wound closure in A549 cells is not due to PBrP-induced growth inhibition, we used the MTT assay (Supplemental Figure 1). The results showed that PBrP did not exert any growth inhibitory effects during 24 h of incubation in the medium containing a low concentration of FBS. Therefore, growth inhibition is not involved in the PBrP-inhibited migration of A549 cells.

### PBrP blocks TGF-β signalling by enhancing degradation of TβRII

As TGF-β receptors play a pivotal role in Smad-mediated signalling and EMT, we investigated whether PBrP regulates TGF-β signalling by altering the TGF-β receptor turnover. Mv1Lu, A549, HepG2 and Clone 9 cells were pretreated with increasing PBrP concentrations or 0.2 μM of PBrP for 0.5–6 h followed by TGF-β stimulation to resemble the conditions used in the experiments of Smad2/3 phosphorylation. We observed that PBrP accelerated TβRII degradation in a dose-dependent manner (Figure 5(A,B)) with an EC_50_ value of 0.05 to 0.1 μM, which was similar to the result of Smad2/3 phosphorylation (Figure 2(A,B)). PBrP (0.2 μM) treatment reduced TβRII protein levels >78% within approximately 4 h in all cell lines (Figure 5(C–F)). At least 2 h were required for PBrP to begin reducing the TβRII levels, with the maximum reduction occurring by 6 h of treatment (Figure 5(C–F)). TβRI protein levels were unaffected by all PBrP concentrations and treatment periods applied (Figure 5(D–F)). To determine whether PBrP enhances exogenous TβRII degradation, Mv1Lu cells transiently expressing TβRII-flag were treated with 0.2 μM of PBrP for increasing periods or with increasing PBrP concentrations for 6 h and were subjected to immunoblotting with an anti-flag antibody. Figure 5(G,H) shows that PBrP-induced TβRII-flag turnover in time- and dose-dependent manners, similar to the endogenous TβRII degradation, observed in several cell lines. These findings suggest that the PBrP-induced TβRII protein reduction is due to increased degradation. To further assess the effect of PBrP on TβRII degradation, we conducted pulse and chase experiments with cycloheximide (CHX) to monitor the stability of endogenous TβRII protein after PBrP treatment. Mv1Lu cells were incubated with 10 μM of CHX in the presence or absence of 0.2 μM of PBrP for 0, 0.5, 2, 4 and 6 h, followed by Western blotting. PBrP caused a rapid decline in the TβRII level, and CHX shortened the half-life of TβRII in CHX-treated cells (Figure 5(I)). In addition, the expression level of TβRII mRNA was evaluated through RT-PCR. The results reveal that the TβRII mRNA levels in response to PBrP treatment are stable during the experimental period (Supplemental Figure 3). Taken together, these results suggest that PBrP impairs TβRII stability and facilitates TβRII degradation. To address whether PBrP reduced the cell surface or total TβRII, we determined the effect of PBrP on the TβRII level on the cell surface through surface protein biotinylation. Biotin-labelled TβRII-flag on the cell surface rapidly and significantly declined faster than TβRII-flag in total cell lysates, when the Mv1Lu cells were exposed to 0.2 μM of PBrP (Figure 5(J)). Immunoblotting for biotin-labelled TβRII in the NeutrAvidin bead pulled-down pellet reveals reduced levels of TβRII on the cell surface 1 h after PBrP treatment (Figure 5(J)). These results suggest that PBrP cleared TβRII from the cell surface and targeted it for degradation. To confirm this observation through immunofluorescent staining, Mv1Lu cells expressing TβRII-flag were treated with PBrP for 1 h, followed by paraformaldehyde fixation and permeabilisation, and then confocal microscopy examination. Before PBrP treatment, TβRII-flag was predominately localised in the plasma membrane (Figure 5(Kc)). After 1 h of incubation with 0.2 μM of PBrP, the amount of TβRII-flag in the plasma membrane decreased and TβRII-flag had accumulated in the perinuclear regions of cells (Figure 5(Kg)). The counterstaining of Rab5 (red) and 4,6-diamidino-2-phenylindole (DAPI, blue) represent the early endosomes and nuclei, respectively.

Figure 5.PBrP treatment resulted in the internalisation and rapid degradation of T*β*RII. Serum-starved Mv1Lu (A) and A549 (B) cells were treated with increasing concentrations of PBrP (0.01–1 *μ*M) for 6 h and then stimulated with 20 or 100 pM of TGF-*β* for 30 min. Cells were analysed through Western blotting for T*β*RII protein levels. Similarly, Mv1Lu (C), A549 (D), HepG2 (E) and Clone 9 (F) cells growing in a low-serum medium were treated with 0.2 or 0.5 *μ*M of PBrP for 0, 1, 2, 4 and 6 h followed by 50 or 100 pM of TGF-*β* stimulation for 30 min. Cell lysates were analysed for T*β*RII, T*β*RI or *β*-actin protein expression through Western blotting. The show results are those of the same samples from Figure 2 (A to G) and share the same *β*-actin for load control. Panels (A to D) show the representative images and illustrate quantitative analyses of ECL (mean ± SD) from at least three independent experiments (% of untreated control); *p < .05, **p < .01. Mv1Lu cells transiently expressing T*β*RII-flag were treated with 0.01–0.2 *μ*M of PBrP for 6 h (G) or 0.2 *μ*M of PBrP for 0, 1, 2, 4 and 6 h (H). Subsequently, the cells lysates were subjected to Western blot analysis using an antibody against the flag tag. The mock showed no signal, which suggested that the flag signal from the T*β*RII-flag was not an artefact. (I) PBrP reduces T*β*RII protein stability. Mv1Lu cells were treated with 0.5 *μ*M of PBrP or a relevant amount of DMSO for the indicated time in the presence or absence of CHX. The amounts of T*β*RII protein from the cell lysates were analysed through Western blotting using anti-T*β*RII and anti-*β*-actin (as a loading control) antibodies. The percentage of the control indicates the T*β*RII amount at each time point relative to the control. (J) PBrP reduced T*β*R-II abundance at the cell surface, as assessed by affinity purification of cell surface proteins using NeutrAvidin-conjugated beads and Western blot. Cells expressing T*β*R-II-flag were pretreated with 0.2 *μ*M PBrP for 0, 1, 2, 3 and 4 h in DMEM containing 0.2% of FBS at 37 °C followed by cell surface biotinylation. NeutrAvidin-precipitated proteins and total lysates were subjected to immunoblotting to detect T*β*R-II-flag. Changes of T*β*R-II abundance were represented as % of control (mean ± SD) from three independent experiments; *p < .05, **p < .01. (K) HEK293 cells co-expressing T*β*RII-flag and Rab5-RFP were treated with 0.2 *μ*M of PBrP for 1 h, followed by fixation and permeabilisation. T*β*RII-flag localisation was visualised by immunofluorescence staining using the anti-flag antibody and Alexa Fluor 488-conjugated secondary antibodies. Bar: 10 *μ*m.

**Figure:**
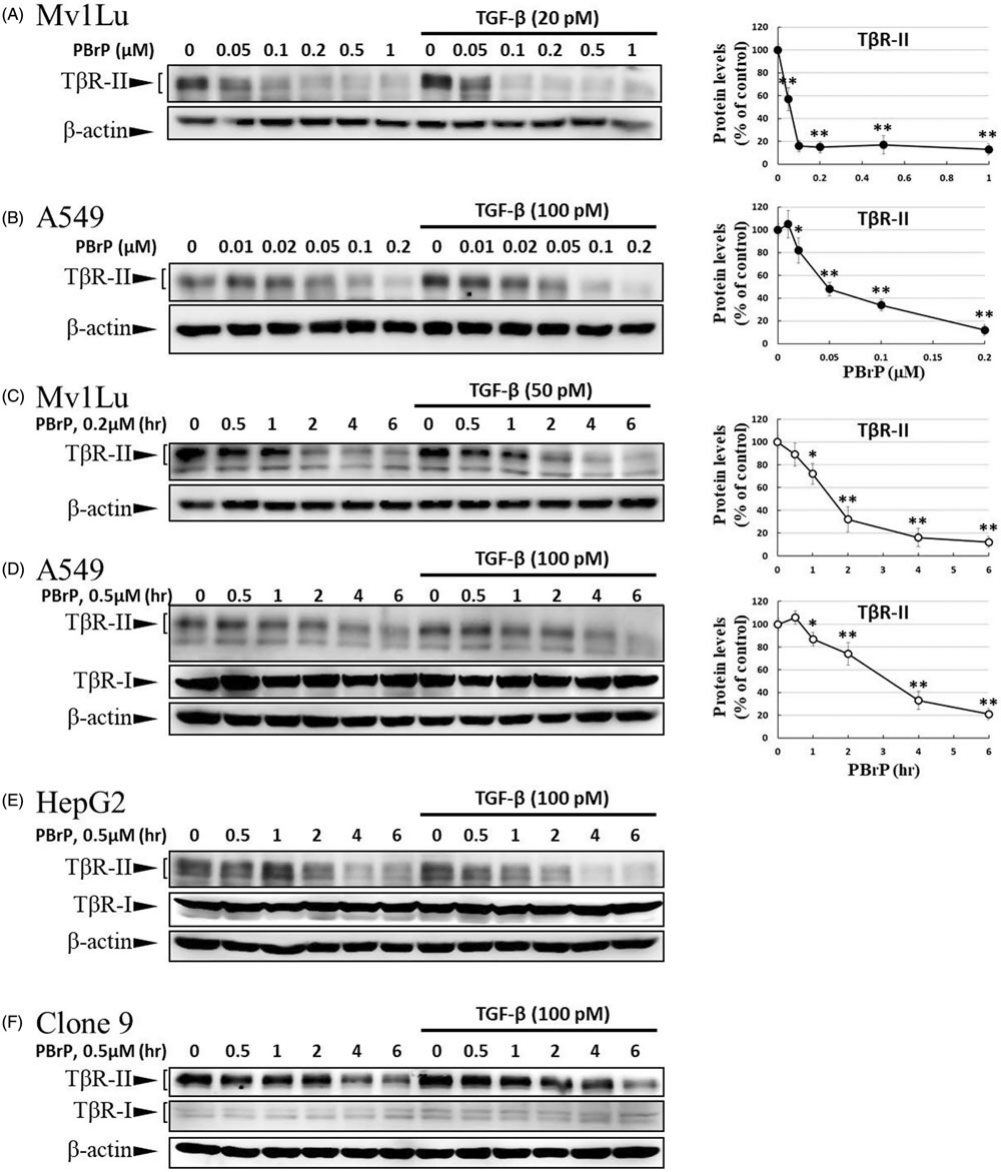

**Figure:**
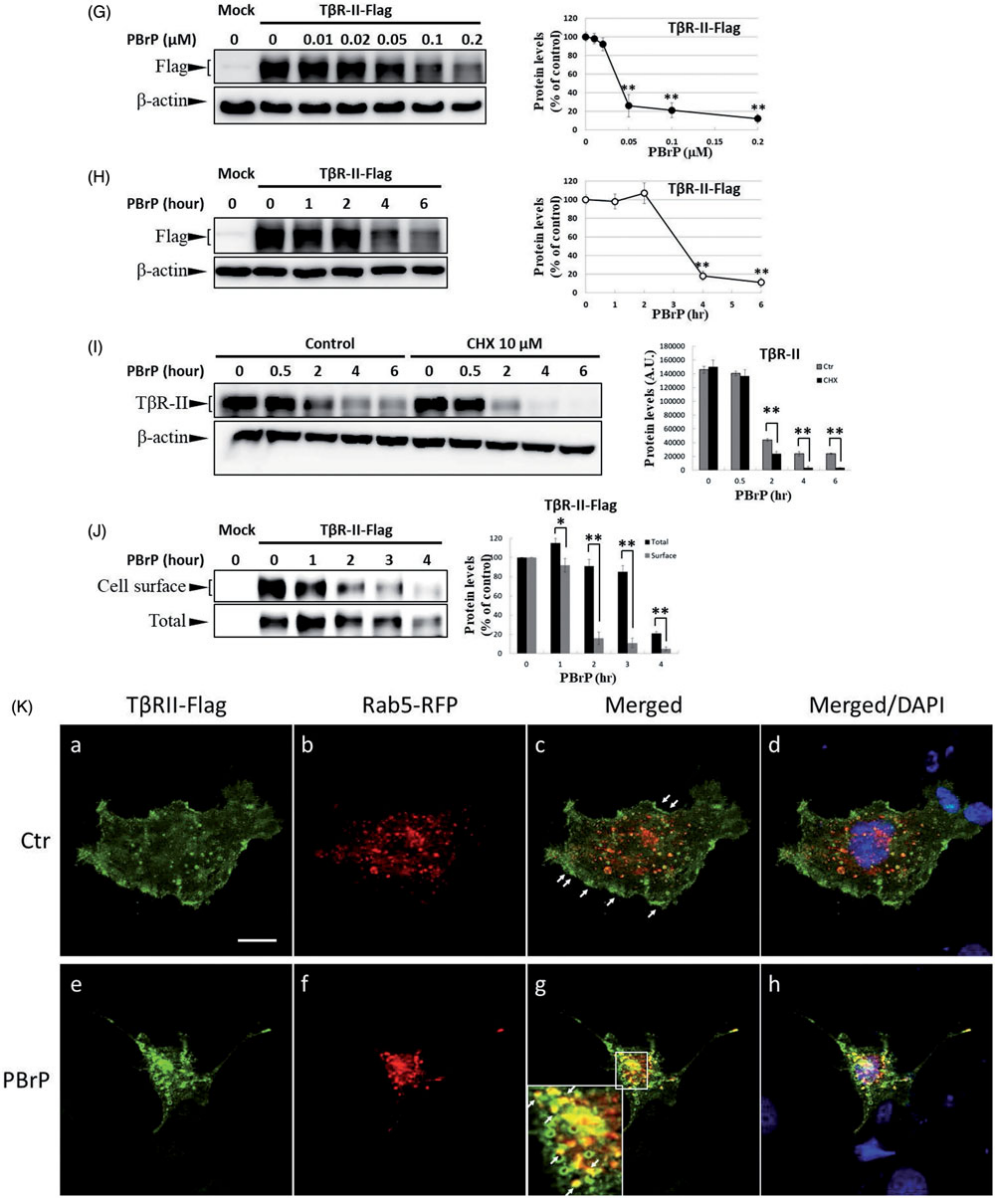

### Repression of Myosin-Va expression by shRNA reduces TβRII levels and TGF-β signalling

**Figure 6.**
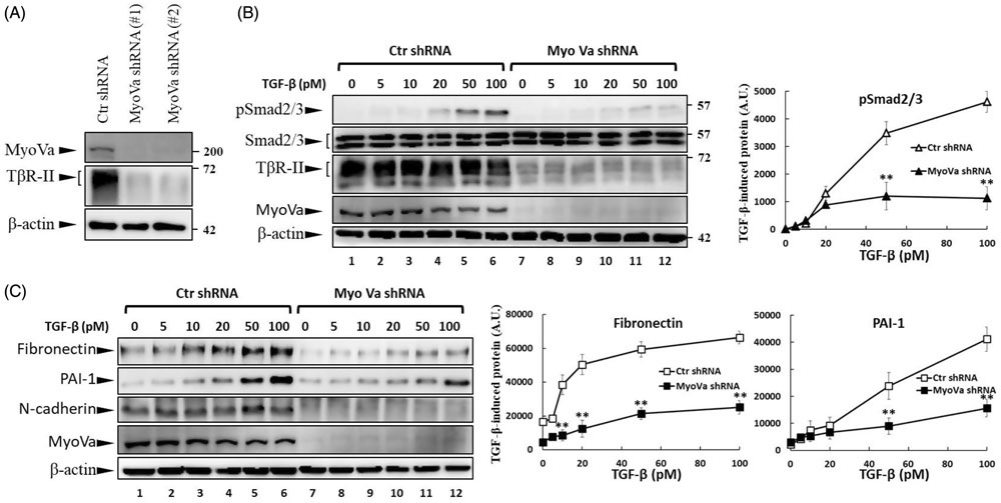
MyoVa depletion enhances T*β*RII turnover and mitigates TGF-*β*/Smad signalling. A549 cells were infected with the lentivirus carrying control (Ctr shRNA) or MyoVa shRNA constructs (MyoVa shRNA#1 and MyoVa shRNA#2). Three days post-infection, total protein was extracted and subjected to Western blot using anti-MyoVa, anti-T*β*RII or anti-*β*-actin antibodies. (A) Two MyoVa shRNAs abolish MyoVa protein production and reduced the T*β*RII protein levels. (B) Effects of MyoVa on TGF-*β*-induced Smad2/3 phosphorylation. A549 cells harbouring MyoVa shRNA#1 or control shRNA were stimulated with 5, 10, 20, 50 and 100 pM of TGF-*β* for 30 min. The amount of pSmad2/3 obtained from the lysates of cells in the absence (Lanes 8–12) or presence (Lanes 1–7) of MyoVa was analysed through Western blotting using anti-pSmad2/3, anti-Smad2/3 and anti-*β*-actin were used as loading control. (C) MyoVa depletion inhibits TGF-*β*-induced fibronectin, PAI-1, and N-cadherin expression. Control and MyoVa-silenced A549 cells were treated with 5, 10, 20, 50 and 100 pM of TGF-*β* for 48 h. The protein abundance of cells in the absence (Lanes 8–12) or presence (Lanes 1–7) of MyoVa was analysed through Western blotting using appropriate antibodies. Right graphs illustrate quantitative analyses of ECL (mean ± SD) from at least three independent experiments; **p < .01.

PBrP is a reversible non-competitive inhibitor of myosin Va (MyoVa). PBrP inhibits ATPase activity by inducing structural changes allosterically, thereby reducing the coordination between the nucleotide and actin-binding sites and causing global changes in myosin dynamics^4^. The inhibitory effects of PBrP on TGF-β signalling prompted us to investigate the functional role of MyoVa in the regulation of TGF-β responsiveness. To study the role of MyoVa in TGF-β signalling, an RNA interference with lentiviral short hairpin RNA (shRNA) was used to suppress MyoVa expression in A549 cells, a cell line that is highly responsive to TGF-β stimulation and expresses a high MyoVa level^26^. A549 cells were separately infected with lentivirus harbouring two sets of MyoVa shRNA, namely shRNA1 and shRNA2, or control shRNA. Western blot results revealed that MyoVa shRNA1 and 2 effectively reduced MyoVa protein levels, manifesting reductions of 98% and 95%, respectively (Figure 6(A)). Concomitantly, the protein levels of TβRII decreased markedly in both shRNA1- and shRNA2-infected cells. To extend our study of the effect of MyoVa on TGF-β responsiveness, we tested TGF-β-induced Smad2/3 phosphorylation and EMT protein production in MyoVa shRNA1-infected cells. Control shRNA and MyoVa shRNA lentivirus-transduced cells were treated with increasing concentrations of TGF-β for 30 min or 48 h to examine TGF-β-induced Smad2/3 phosphorylation and EMT protein production, respectively. Western blot analysis revealed that cells with MyoVa shRNA exhibited considerably lower TβRII and pSmad2/3 levels than those cells harbouring control shRNA with or without TGF-β stimulation (Figure 6(B)). In addition, MyoVa-silenced cells exhibited low fibronectin and low PAI-1 levels (Figure 6(C)) upon TGF-β stimulation, which was consistent with the results of TGF-β-induced Smad2/3 phosphorylation. These results suggest that the inhibition of MyoVa leads to the substantial downregulation of TβRII protein levels, resulting in the inhibition of TGF-β responsiveness. This effect is similar to that of PBrP, which is likely mediated by its effect on the subcellular trafficking of TβRII.

### Late endosome-lysosome pathway is essential for PBrP-induced TβRII degradation

**Figure 7.**
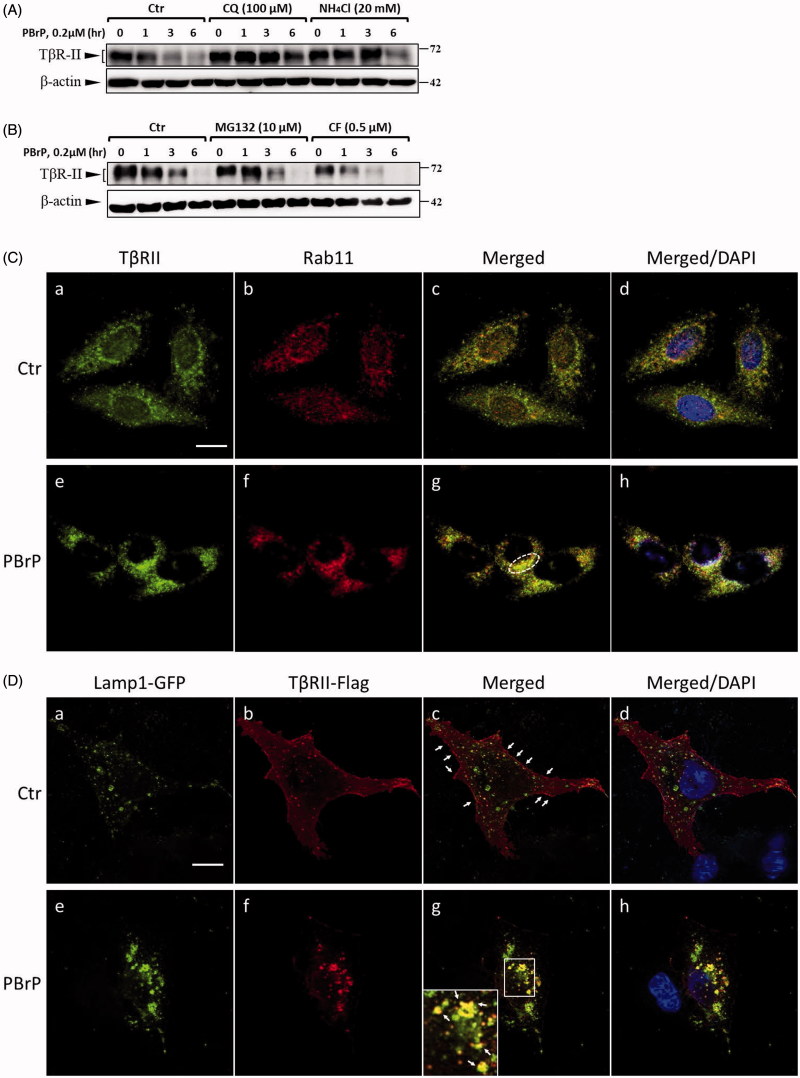
PBrP induces T*β*RII turnover through the late endosome–lysosome pathway and is impaired by lysosome inhibitors. Mv1Lu cells grown in 0.2% of FBS-containing DMEM were incubated with PBrP (0.5 *μ*M) with chloroquine (100 *μ*M) or NH_4_Cl (20 mM) (A) for 1, 3 and 6 h or MG132 (20 *μ*M) and carfilzomib (0.5 *μ*M) (B). Subsequently, the cell lysates were subjected to SDS-PAGE, and T*β*RII expression was analysed through Western blotting and quantified through densitometry. (C) T*β*RII was enriched in the Rab11-positive endosomal compartment in PBrP-treated cells. Mv1Lu cells were treated with 0.5 *μ*M of PBrP for 1 h in low-serum DMEM. Endogenous T*β*RII and Rab11 were visualised through immunofluorescence staining using Alexa Fluor 488- and 594-conjugated secondary antibodies, respectively. (D) T*β*RII was internalised and directed into lysosomes in PBrP-treated cells. HEK293 cells transiently co-expressing T*β*RII-flag and Lamp-1-GFP were treated with 0.5 *μ*M of PBrP for 2.5 h in low-serum DMEM. After fixation and permeabilisation, T*β*RII-flag was visualised through immunofluorescence staining using anti-flag antibodies and an Alexa Fluor 594-conjugated secondary antibody. PBrP reduced membrane-associated T*β*RII-flag (red) and increased co-localisation with the lysosome marker Lamp-1 (green). Bar: 10 *μ*m.

Because PBrP promotes TβRII degradation, TGF-β receptor turnover is considered to be mediated by the lysosome and proteasome. To determine whether PBrP-induced TβRII degradation occurred through the lysosomal pathway in this study, we assessed TβRII turnover in the presence of two inhibitors, namely CQ and ammonium chloride (NH_4_Cl), which are lysosomotropic agents that prevent endosomal acidification and lysosomal degradation. CQ and NH_4_Cl significantly attenuated PBrP-induced TβRII degradation (Figure 7(A)), suggesting that the PBrP-induced degradation of TβRII is impaired by lysosome inhibitors. To determine whether the proteasome pathway is involved in TβRII degradation, we employed proteasome inhibitors MG132 and carfilzomib, both of which inhibit the chymotrypsin-like activity of the 20S proteasome. Neither treatment reversed PBrP-induced degradation (Figure 7(B)). These results indicate that the PBrP-induced TβRII degradation is mediated by the lysosomal pathway rather than the proteasome pathway. Accelerated receptor degradation might be due to receptor accumulation in the intracellular compartment, which is directed toward degradation, for example through the late endosome–lysosome route. To confirm this concept, we examined the receptor internalisation and colocalisation with Rab11- or lysosome-associated membrane glycoprotein 1 (Lamp-1)-positive vesicles in cells. Confocal immunofluorescence microscopy was conducted to assess the colocalisation of endogenous TβRII and Rab11 in Mv1Lu cells. Figure 7(C) shows that under control, a large portion of TβRII exhibited typical punctate vesicle structures and was scattered throughout the plasma membrane and cytoplasm. However, upon addition of PBrP, endogenous TβRII was localised mainly in perinuclear cytoplasmic inclusions (Figure 7(Ce)) and a high level of TβRII was co-localised with Rab11 (Figure 7(Cg)). Figure 7(D) shows that in HEK293 cells, overexpressed TβRII-flag was primarily located on the cell surface (Figure 7(Db)). PBrP treatment for 2.5 h profoundly reduced the TβRII-flag levels on the cell surface (Figure 7(Df)) and enhanced Lamp-1-GFP colocalisation near the nuclei (Figure 7(Dg)). These results suggest that PBrP promotes the TβRII degradation through a late endosome–lysosome pathway.

### PBrP blocks TGF-β signalling by enhancing degradation of TβRII via caveolae

**Figure 8.**
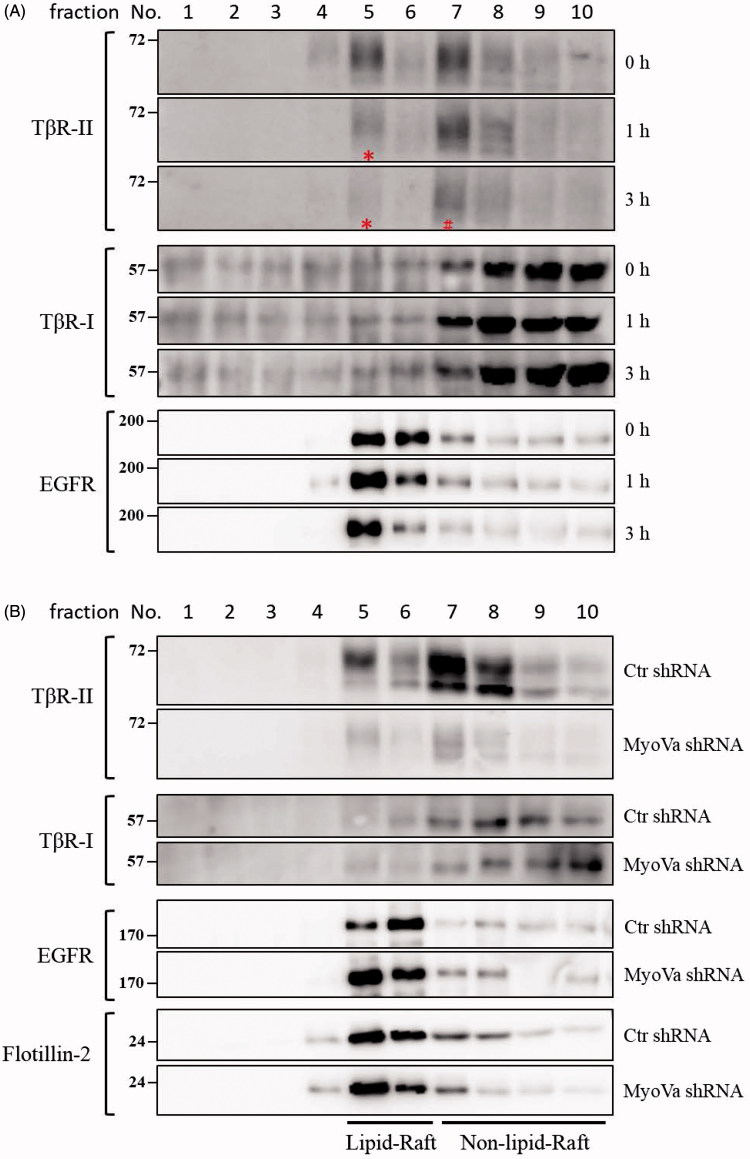
PBrP induces T*β*RII degradation in lipid rafts. A549 cells or shRNA-silenced A549 cells were left untreated or incubated with 0.5 *μ*M of PBrP for 0, 1 and 3 h in low-serum DMEM and lysed. Subsequently, 10 sucrose density gradient fractions of the lysates were collected through ultracentrifugation, as described in *Materials and methods* section. Thirty microgram of protein from each fraction was subjected to SDS-PAGE and transferred onto PVDF membranes, and blotted with anti-T*β*RII, anti-T*β*RI and EGFR antibodies. Protein samples from various treatments in acrylamide gel strips were synchronously electrotransferred onto the same PVDF membrane and then simultaneously proceeded for Western blotting; therefore, the signals of distinct proteins from various treatments were mutually comparable. (A) PBrP treatment reduced T*β*RII predominately in raft fractions within 3 h. (B) MyoVa depletion reduced T*β*RII in raft and non-raft fractions without altering the protein abundance and subcellular compartmentation of T*β*RI, EGFR or raft marker flotillin-2.

The regulation of TGF-β receptor signalling has been linked to two distinct endocytic pathways. Caveolae-mediated endocytosis directs receptors into late endosomes en route to lysosomes for degradation, whereas clathrin-mediated endocytosis sends receptors to early endosomes, promotes sustained signalling and allows the recycling of receptors back to the cell surface^10^^,^^27–29^. We have used subcellular density gradient fractionation to observe the effect of PBrP on TβRII compartmentation between lipid raft and lipid non-raft fractions in chronological order. A549 cells were treated with 0.2 μM of PBrP for 0, 1 and 3 h and then subjected to sucrose gradient ultracentrifugation. The resulting fractions were analysed through Western blot analysis. As shown in Figure 8(A), TβRII was equally distributed in lipid raft and lipid non-raft fractions in the control (0 h) experiment. After the first hour of treatment (1 h), PBrP strongly reduced raft-associated TβRII (in fraction 5, marked with a star sign) without altering the TβRII level in non-raft fractions (fraction 7). By contrast, the progressive decrease in TβRII abundance in non-raft fractions started within 3 h of PBrP exposure (Figure 8(A), in fraction 7, marked with a pound sign). This result suggests that TβRII is internalised and further degraded through caveolin-rich lipid rafts. PBrP treatment did not change the abundance and inter-domain partitioning of other receptors such as TβRI and EGFR, suggesting that PBrP-induced intracellular translocation and protein turnover are exclusive for TβRII. Western immunoblot analyses of the serial samples of subcellular density gradient fractions from A549 cells infected with lentivirus harbouring MyoVa shRNA exhibited a considerable reduction in lipid-raft-associated TβRII (Figure 8(B)). By contrast, the protein levels and intracellular distribution of TβRI, EGFR and caveolin-1 did not change in MyoVa-silenced cells.

## Discussion

TGF-β is a cytokine that regulates a wide range of cellular functions among normal and pathological processes. In advanced cancers, TGF-β promotes tumour-growth and contributes to the hallmarks of cancer, including EMT, cell-growth, metastasis, angiogenesis, inflammation and evasion from immune surveillance. Several pharmacological approaches have been used to prevent TGF-β signalling, including small-molecule inhibitors, antisense oligonucleotides and monoclonal antibodies. Small-molecule inhibitors of TGF-β signalling are valuable as scientific tools for understanding TGF-β-mediated biological processes and can be utilised as leads for developing therapeutic agents to treat many severe diseases associated with TGF-β malfunction^30^.

This study clearly demonstrates that PBrP represses TGF-β-stimulated responsiveness and EMT. Immunofluorescence and Western blot analyses revealed that TGF-β-induced Smad2/3 phosphorylation and nuclear translocation are inhibited by PBrP treatment in a concentration-dependent manner. The induction of EMT-related genes such as fibronectin, vimentin, N-cadherin and *PAI-1* are markedly inhibited by PBrP treatment in epithelial cells. Additionally, PBrP inhibited TGF-β-increased cell mobility in a cell migration assay. A key finding of this investigation is that PBrP abrogated TGF-β signalling by promoting the degradation of TβRII. TGF-β receptor abundance and the ratio of cell surface to cytoplasm are dynamically regulated by endocytosis and exocytosis, intracellular trafficking, recycling, lysosome degradation and direct proteasomal degradation^10^^,^^28^^,^^31^. Two distinct pathways, namely clathrin-mediated endocytosis and caveolae-mediated internalisation, mediate the internalisation of TGF-β receptors from the cell surface into intracellular compartments^27^. Clathrin-mediated endocytosis facilitates and sustains signalling by recruiting SARA, which facilitates the effective phosphorylation of R-Smad proteins. In addition, caveolae-mediated internalisation directs receptors into the proteasome or lysosome for degradation, and further terminates signalling^32–34^. Our results suggest that PBrP does not promote the proteasomal degradation of TβRII, because carfilzomib and MG132 did not reverse the receptor degradation (Figure 7(B)). Instead, PBrP promotes the lysosomal degradation of receptors because CQ and NH_4_Cl prevent receptor degradation by targeting the intracellular trafficking and recycling of TβRII (Figure 7(A)). However, many studies have documented equal degradation of TβRI and TβRII through the proteasome or lysosome^10^. Our PBrP result is distinct from this mechanism, because TβRII levels changed but TβRI levels did not. In addition, we found no studies on biological reagents or small molecules that dissociate TβRI and TβRII in late endosome–lysosome trafficking. Thus, we propose that there may be an alternative pathway for the specific degradation of TβRII induced by PBrP. Distinct effects of PBrP on TβRI and TβRII degradation are consistent with the idea that specific degradation processes may exist to clear these receptors from the cell surface. In addition, PBrP may serve as a novel chemical probe to study TGF-β signalling and to explore the role of the intracellular compartmentalisation of TβRII in cellular functions as well as the molecular basis of diseases. Our previous study showed that pentabromophenol (PBP), a widely used flame retardant, suppresses TGF-β signalling in a similar fashion as PBrP does. However, PBP promotes TβRII degradation by dislocating TβRII from lipid-raft into proteasome for degradation^21^.

Although the direct target of PBrP remains to be elucidated and PBrP might directly bind TβRII to drive its internalisation and degradation, several studies have reported that PBrP is a potent and reversible inhibitor of MyoVa^5^^,^^35^^,^^16^. Kinetic and structural studies have shown that PBrP is a non-competitive inhibitor that hampers MyoVa function by hindering the ATPase cycle (IC_50_ = 1.2 μM)^3^^,^^16^. More crucially, the inhibition of myosin V activity has exhibited increasing strength in cellular studies, with an IC_50_ value of <0.5 μM, which is close to the concentration used to inhibit TGF-β signalling^5^^,^^35^. Myosins are actin-dependent motor proteins that perform a variety of diverse tasks, including the movement of intracellular molecules and organelles, maintenance of cell adhesion, cytokinesis and muscle contraction. In mammals, the myosin V family has three members, namely Va, Vb and Vc. MyoVa is best characterised by its enzymatic and mechanical properties and intracellular roles^36^^,^^37^. MyoVa has been strongly linked to various stages of exocytosis, namely the capturing, tethering and transport of secretory vesicles approaching the plasma membrane via the actin cytoskeleton^38^^,^^39^. We hypothesise that PBrP blocks the MyoVa-mediated transportation of the exocytic vesicle to the plasma membrane, inhibits TβRII recycling to the cell surface and causes TβRII accumulation and degradation. We used shRNA silencing approaches to determine whether MyoVa can function in relation to TβRII turnover, as well as TGF-β-stimulated cellular responsiveness. In this study, we have confirmed that A549 cells infected with lentivirus harbouring MyoVa shRNA expressed a low level of TβRII without altering TβRI. However, the cells expressing control shRNA exhibited normal abundance of TβRI and TβRII. PBrP-treated cells and MyoVa knockdown cells exhibited similar tendencies in terms of the intracellular compartmentalisation of TβRII. The subcellular density gradient fractionation revealed that PBrP treatment promoted TβRII degradation in lipid-raft membrane fractions in the early stage (1 h), and long-term treatment further abrogated the TβRII level in the lipid non-raft membrane fractions. MyoVa depletion resulted in TβRII degradation primarily in lipid-raft membrane fractions and left a trace amount of TβRII in the lipid non-raft fraction. MyoVa depletion reduced TβRII and coincided with the decreased expression of pSmad2/3, fibronectin, PAI-1 and N-cadherin proteins visualised through Western blot analysis. Although the high efficiency (with a very low IC_50_) of PBrP and the effects of MyoVa shRNA knockdown in TβRII degradation suggested a strong relevance of MyoVa in the regulation of TβRII turnover and TGF-β signalling, we cannot rule out the possibility of the off-target effects of PBrP on other myosins or non-myosins within the cell. By exploring the precise targets of PBrP in TβRII intracellular trafficking, future studies may help us to understand the role of myosins in TGF-β signalling. In addition, because myosins likely act cooperatively with overlapping functions, conducting further biochemical analysis on the joint cellular functions of myosin motors in TGF-β signalling could prove indispensable.

## Conclusion

We conclude that small-molecule inhibitors such as PBrP that target vesicle trafficking and inhibit TGF-β signalling could be developed into a broad-spectrum therapeutic agent to cure cancer and fibrosis diseases. Although targeting motor proteins with small-molecule inhibitors therapeutically to treat TGF-β-related diseases is attractive, targeting fundamental processes such as endosomal trafficking may eventually prove to be problematic because such compounds may be intrinsically toxic to the whole organism. In addition, the multifunctional and pleiotropic effects of TGF-β and its role in cell proliferation, tissue homeostasis, and immunity raise concerns regarding potential adverse effects, which must be considered when inducing TGF-β signalling abrogation. Accordingly, although this approach is still out on such therapeutic applications, the value of such chemical probes has been clearly illustrated previously and will continue to be.

## Supplementary Material

IENZ_1465416_Supplementary_Material.zip

## References

[1] Burkholder PR, Pfister RM, Leitz FH. Production of a pyrrole antibiotic by a marine bacterium. Appl Microbiol 1966;14:649–53. 4380876 10.1128/am.14.4.649-653.1966 PMC546803

[2] Ohri RV, Radosevich AT, Hrovat KJ, et al A Re(V)-catalyzed C-N bond-forming route to human lipoxygenase inhibitors. Org Lett 2005;7:2501–4. 15932233 10.1021/ol050897a

[3] Preller M, Chinthalapudi K, Martin R, et al Inhibition of Myosin ATPase activity by halogenated pseudilins: a structure-activity study. J Med Chem 2011;54:3675–85. 21534527 10.1021/jm200259f

[4] Martin R, Jäger A, Böhl M, et al Total synthesis of pentabromo- and pentachloropseudilin, and synthetic analogues–allosteric inhibitors of myosin ATPase. Angew Chem Int Ed Engl 2009;48:8042–6. 19739175 10.1002/anie.200903743

[5] Fedorov R, Böhl M, Tsiavaliaris G, et al The mechanism of pentabromopseudilin inhibition of myosin motor activity. Nat Struct Mol Biol 2009;16:80–8. 19122661 10.1038/nsmb.1542

[6] Miaczynska M, Pelkmans L, Zerial M. Not just a sink: endosomes in control of signal transduction. Curr Opin Cell Biol 2004;16:400–6. 15261672 10.1016/j.ceb.2004.06.005

[7] Hanyaloglu AC, von Zastrow M. Regulation of GPCRs by endocytic membrane trafficking and its potential implications. Annu Rev Pharmacol Toxicol 2008;48:537–68. 18184106 10.1146/annurev.pharmtox.48.113006.094830

[8] Scita G, Di Fiore PP. The endocytic matrix. Nature 2010;463:464–73. 20110990 10.1038/nature08910

[9] Hu Y, Chuang JZ, Xu K, et al SARA, a FYVE domain protein, affects Rab5-mediated endocytosis. J Cell Sci 2002;115:4755–63. 12432064 10.1242/jcs.00177 PMC3899687

[10] Di Guglielmo GM, Le Roy C, Goodfellow AF, Wrana JL. Distinct endocytic pathways regulate TGF-beta receptor signalling and turnover. Nat Cell Biol 2003;5:410–21. 12717440 10.1038/ncb975

[11] Mitchell H, Choudhury A, Pagano RE, Leof EB. Ligand-dependent and -independent transforming growth factor-beta receptor recycling regulated by clathrin-mediated endocytosis and Rab11. Mol Biol Cell 2004;15:4166–78. 15229286 10.1091/mbc.E04-03-0245 PMC515349

[12] Chen CL, Hou WH, Liu IH, et al Inhibitors of clathrin-dependent endocytosis enhance TGFbeta signaling and responses. J Cell Sci 2009;122:1863–71. 19461075 10.1242/jcs.038729 PMC2684837

[13] Huang SS, Liu IH, Chen CL, et al 7-Dehydrocholesterol (7-DHC), but not cholesterol, causes suppression of canonical TGF-beta signaling and is likely involved in the development of atherosclerotic cardiovascular disease (ASCVD). J Cell Biochem 2017;118:1387–400. 27862220 10.1002/jcb.25797 PMC6123222

[14] Chen CL, Tetri LH, Neuschwander-Tetri BA, et al A mechanism by which dietary trans fats cause atherosclerosis. J Nutr Biochem 2011;22:649–55. 21036587 10.1016/j.jnutbio.2010.05.004 PMC3125015

[15] Chen CL, Chen YP, Lin MW, et al Euphol from Euphorbia tirucalli negatively modulates TGF-beta responsiveness via TGF-beta receptor segregation inside membrane rafts. PLoS One 2015;10:e0140249. 26448474 10.1371/journal.pone.0140249 PMC4598150

[16] Martin R, Risacher C, Barthel A, et al Silver(I)-catalyzed route to pyrroles: synthesis of halogenated pseudilins as allosteric inhibitors for myosin ATPase and X-ray crystal structures of the protein-inhibitor complexes. Eur J Org Chem 2014;21:4487–505.

[17] Huang SS, Chen CL, Huang FW, et al DMSO enhances TGF-β activity by recruiting the type II TGF-β receptor from intracellular vesicles to the plasma membrane. J Cell Biochem 2016;117:1568–79. 26587792 10.1002/jcb.25448 PMC6123219

[18] Poncelet AC, de Caestecker MP, Schnaper HW. The transforming growth factor-beta/SMAD signaling pathway is present and functional in human mesangial cells. Kidney Int 1999;56:1354–65. 10504488 10.1046/j.1523-1755.1999.00680.x

[19] Cobbs SL, Gooch JL. NFATc is required for TGFbeta-mediated transcriptional regulation of fibronectin. Biochem Biophys Res Commun 2007;362:288–94. 17719012 10.1016/j.bbrc.2007.07.186 PMC2083570

[20] Widom RL, Culic I, Lee JY, Korn JH. Cloning and characterization of hcKrox, a transcriptional regulator of extracellular matrix gene expression. Gene 1997;198:407–20. 9370309 10.1016/s0378-1119(97)00360-0

[21] Chen CL, Yang PH, Kao YC, et al Pentabromophenol suppresses TGF-beta signaling by accelerating degradation of type II TGF-beta receptors via caveolae-mediated endocytosis. Sci Rep 2017;7:43206. 28230093 10.1038/srep43206 PMC5322341

[22] Chen CL, Kao YC, Yang PH, et al A small dibromotyrosine derivative purified from *Pseudoceratina* Sp. suppresses TGF-β responsiveness by inhibiting TGF-β type I receptor serine/threonine kinase activity. J Cell Biochem 2016;117:2800–14. 27153151 10.1002/jcb.25581

[23] Chen CL, Liu IH, Fliesler SJ, et al Cholesterol suppresses cellular TGF-beta responsiveness: implications in atherogenesis. J Cell Sci 2007;120:3509–21. 17878231 10.1242/jcs.006916 PMC2045688

[24] Zhao B, Wang Q, Du J, et al PICK1 promotes caveolin-dependent degradation of TGF-β type I receptor. Cell Res 2012;22:1467–78. 22710801 10.1038/cr.2012.92 PMC3463259

[25] Ozdamar B, Bose R, Barrios-Rodiles M, et al Regulation of the polarity protein Par6 by TGFbeta receptors controls epithelial cell plasticity. Science 2005;307:1603–9. 15761148 10.1126/science.1105718

[26] Lecuona E, Minin A, Trejo HE, et al Myosin-Va restrains the trafficking of Na+/K+-ATPase-containing vesicles in alveolar epithelial cells. J Cell Sci 2009;122:3915–22. 19808891 10.1242/jcs.046953 PMC2773192

[27] Le Roy C, Wrana JL. Clathrin- and non-clathrin-mediated endocytic regulation of cell signalling. Nat Rev Mol Cell Biol 2005;6:112–26. 15687999 10.1038/nrm1571

[28] Chen YG. Endocytic regulation of TGF-beta signaling. Cell Res 2009;19:58–70. 19050695 10.1038/cr.2008.315

[29] Lonn P, Moren A, Raja E, et al Regulating the stability of TGF beta receptors and Smads. Cell Res 2009;19:21–35. 19030025 10.1038/cr.2008.308

[30] Chung CL, Wang SW, Martin R, et al Pentachloropseudilin inhibits transforming growth factor-beta (TGF-beta) activity by accelerating cell-surface type II TGF-beta receptor turnover in target cells. ChemBioChem 2018;19:851–64. 29369495 10.1002/cbic.201700693

[31] Willems E, Cabral-Teixeira J, Schade D, et al Small molecule-mediated TGF-beta type II receptor degradation promotes cardiomyogenesis in embryonic stem cells. Cell Stem Cell 2012;11:242–52. 22862949 10.1016/j.stem.2012.04.025 PMC3419596

[32] Razani B, Zhang XL, Bitzer M, et al Caveolin-1 regulates transforming growth factor (TGF)-beta/SMAD signaling through an interaction with the TGF-beta type I receptor. J Biol Chem 2001;276:6727–38. 11102446 10.1074/jbc.M008340200

[33] Penheiter SG, Mitchell H, Garamszegi N, et al Internalization-dependent and -independent requirements for transforming growth factor beta receptor signaling via the Smad pathway. Mol Cell Biol 2002;22:4750–9. 12052882 10.1128/MCB.22.13.4750-4759.2002 PMC133902

[34] Hayes S, Chawla A, Corvera S. TGF beta receptor internalization into EEA1-enriched early endosomes: role in signaling to Smad2. J Cell Biol 2002;158:1239–49. 12356868 10.1083/jcb.200204088 PMC2173232

[35] Bond LM, Tumbarello DA, Kendrick-Jones J, Buss F. Small-molecule inhibitors of myosin proteins. Future Med Chem 2013;5:41–52. 23256812 10.4155/fmc.12.185 PMC3971371

[36] Sellers JR, Veigel C. Walking with myosin V. Curr Opin Cell Biol 2006;18:68–73. 16378722 10.1016/j.ceb.2005.12.014

[37] Vale RD. Myosin V motor proteins: marching stepwise towards a mechanism. J Cell Biol 2003;163:445–50. 14610051 10.1083/jcb.200308093 PMC2173644

[38] Rudolf R, Bittins CM, Gerdes HH. The role of myosin V in exocytosis and synaptic plasticity. J Neurochem 2011;116:177–91. 21077886 10.1111/j.1471-4159.2010.07110.x

[39] Bond LM, Brandstaetter H, Sellers JR, et al Myosin motor proteins are involved in the final stages of the secretory pathways. Biochem Soc Trans 2011;39:1115–19. 21936774 10.1042/BST0391115 PMC3403808

