# Supplementary material for: Pentabromopseudilin: a myosin V inhibitor suppresses TGF-β activity by recruiting the type II TGF-β receptor to lysosomal degradation
Source: J Enzyme Inhib Med Chem. 2018 May 16;33(1):920–35. doi: 10.1080/14756366.2018.1465416 (PMC6009923; doi:10.1080/14756366.2018.1465416)
Supplement: IENZ_1465416_Supplementary_Material.zip [file IENZ_A_1465416_SM1500.zip › IENZ_1465416_Supplementary Material.pptx]

## Slide 1
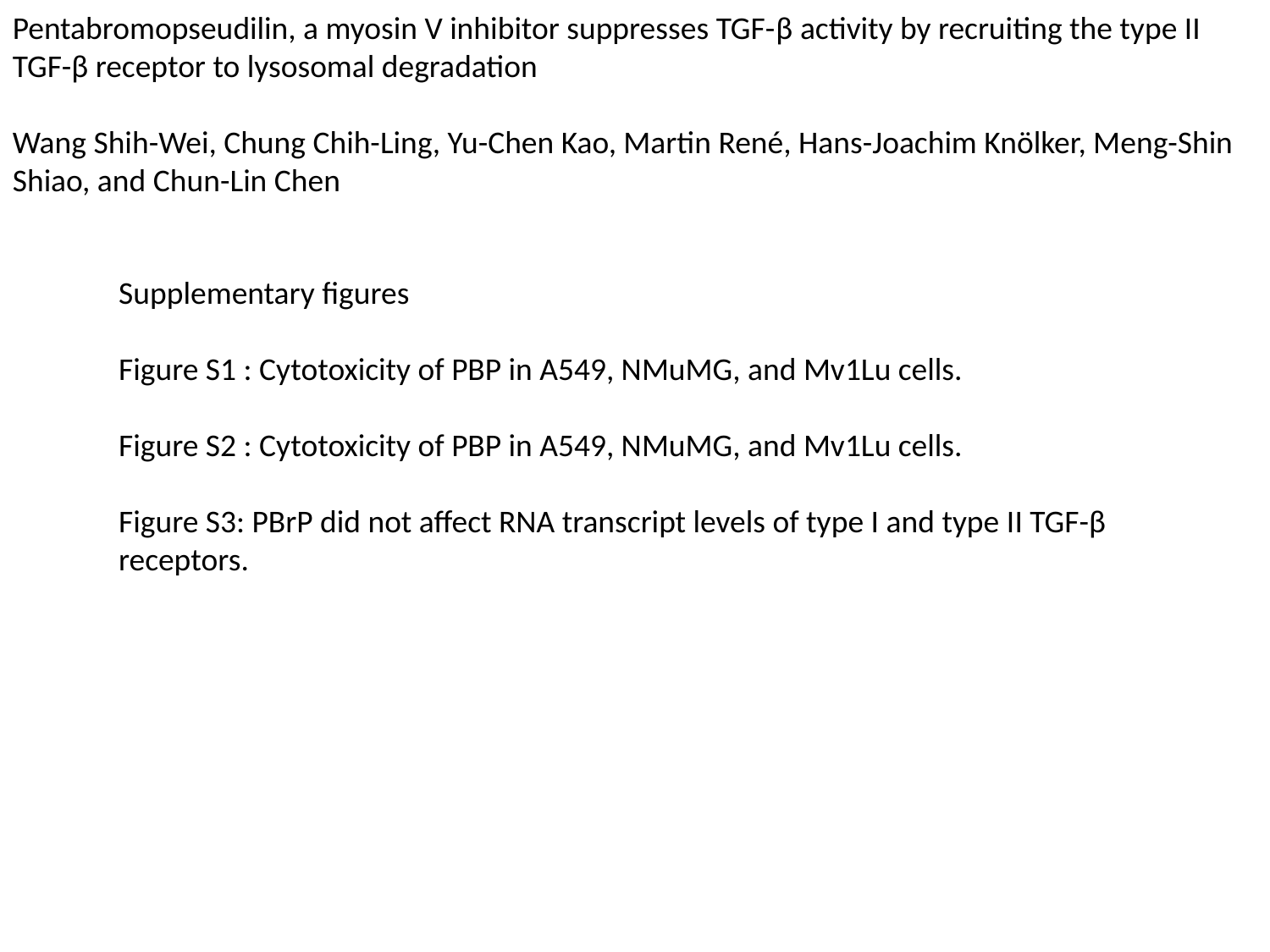

Pentabromopseudilin, a myosin V inhibitor suppresses TGF-β activity by recruiting the type II TGF-β receptor to lysosomal degradation
Wang Shih-Wei, Chung Chih-Ling, Yu-Chen Kao, Martin René, Hans-Joachim Knölker, Meng-Shin Shiao, and Chun-Lin Chen
Supplementary figures
Figure S1 : Cytotoxicity of PBP in A549, NMuMG, and Mv1Lu cells.
Figure S2 : Cytotoxicity of PBP in A549, NMuMG, and Mv1Lu cells.
Figure S3: PBrP did not affect RNA transcript levels of type I and type II TGF-β receptors.

## Slide 2
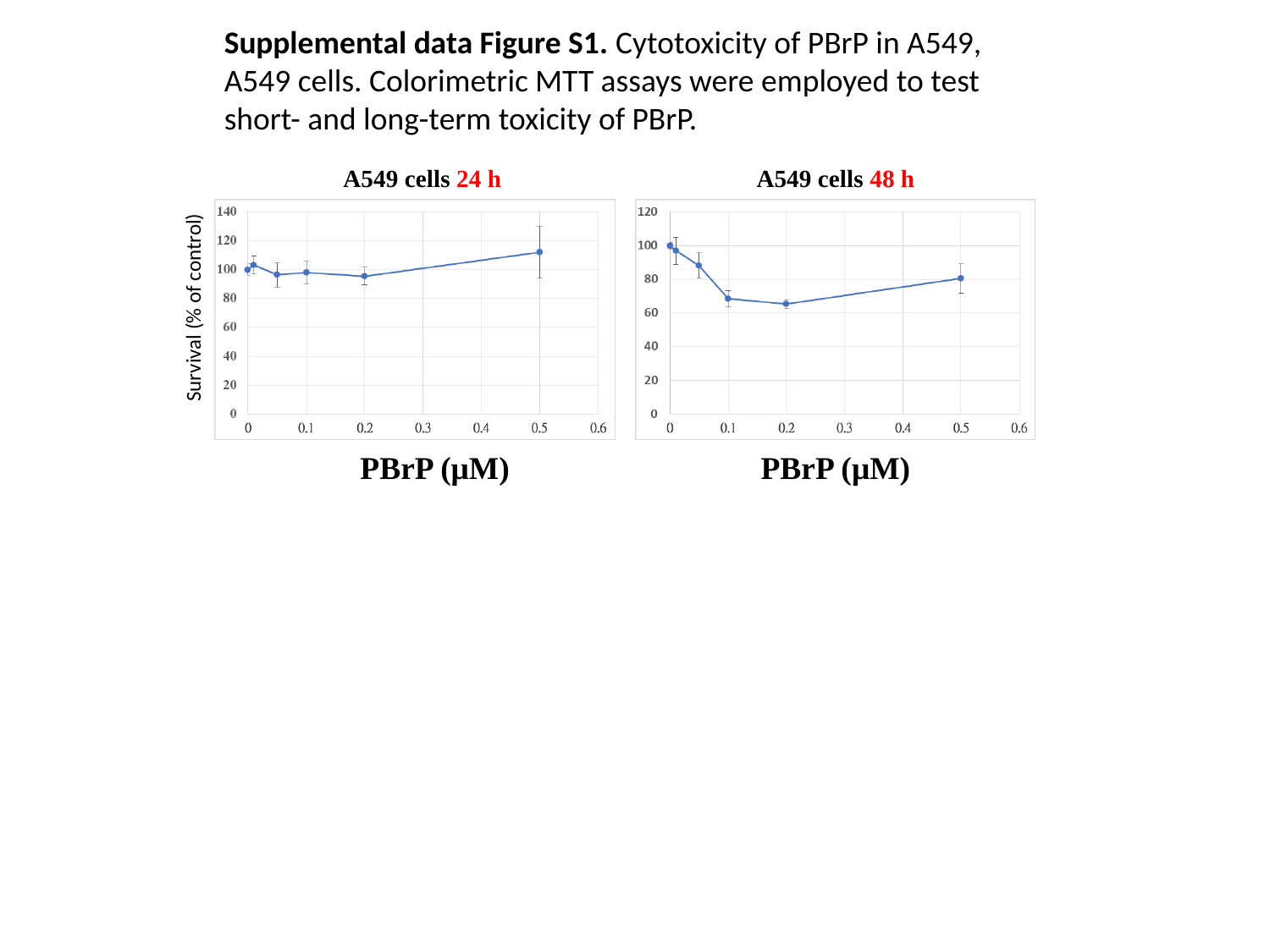

Supplemental data Figure S1. Cytotoxicity of PBrP in A549, A549 cells. Colorimetric MTT assays were employed to test short- and long-term toxicity of PBrP.
A549 cells 24 h
A549 cells 48 h
Survival (% of control)
PBrP (μM)
PBrP (μM)

## Slide 3
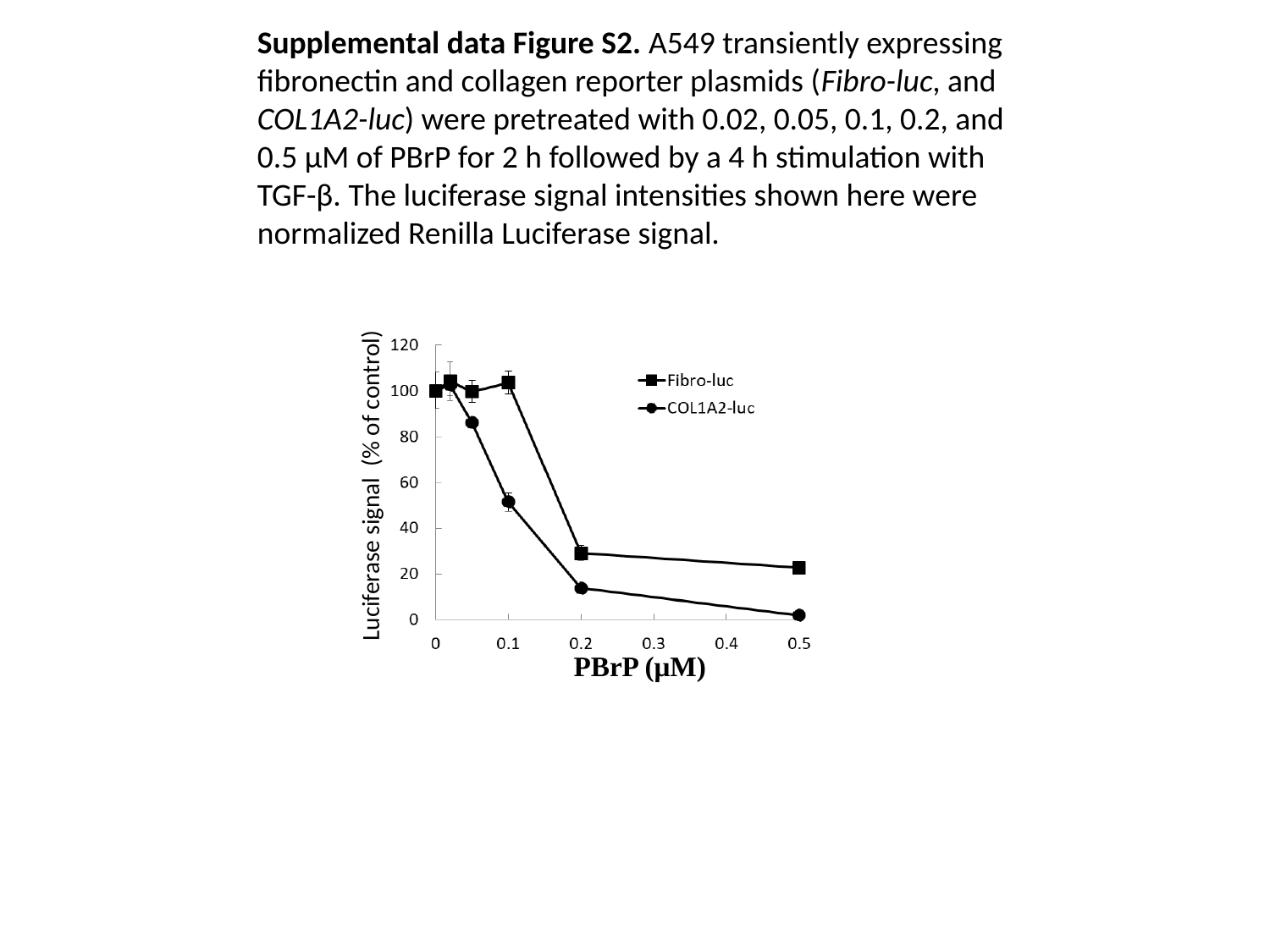

Supplemental data Figure S2. A549 transiently expressing fibronectin and collagen reporter plasmids (Fibro-luc, and COL1A2-luc) were pretreated with 0.02, 0.05, 0.1, 0.2, and 0.5 μM of PBrP for 2 h followed by a 4 h stimulation with TGF-β. The luciferase signal intensities shown here were normalized Renilla Luciferase signal.
Luciferase signal (% of control)
PBrP (μM)

## Slide 4
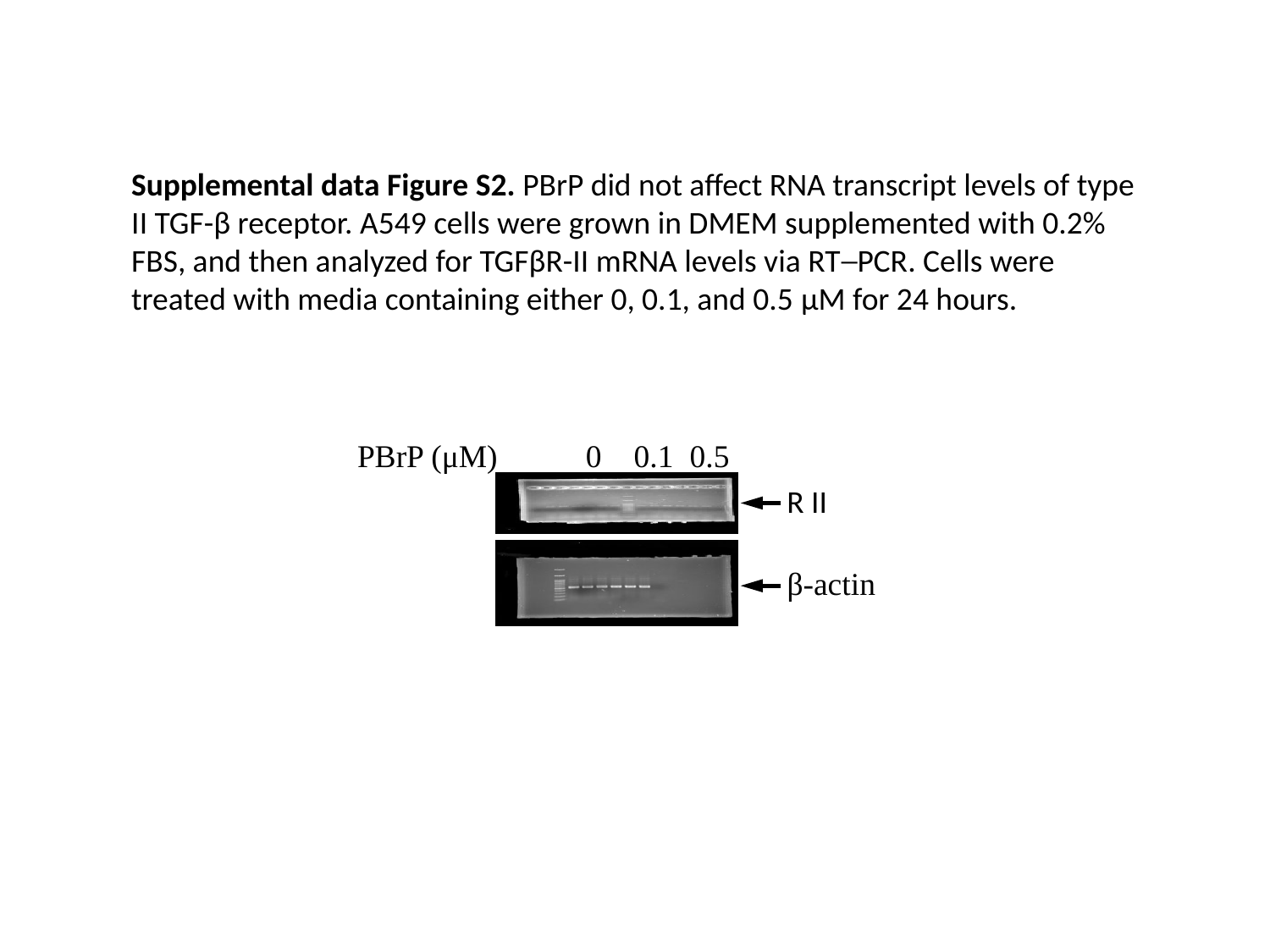

Supplemental data Figure S2. PBrP did not affect RNA transcript levels of type II TGF-β receptor. A549 cells were grown in DMEM supplemented with 0.2% FBS, and then analyzed for TGFβR-II mRNA levels via RT─PCR. Cells were treated with media containing either 0, 0.1, and 0.5 μM for 24 hours.
PBrP (μM) 0 0.1 0.5
R II
β-actin
